# Molecularly imprinted ‘traps’ for sulfonylureas prepared using polymerisable ion pairs†

**DOI:** 10.1039/c8ra01135d

**Published:** 2018-04-17

**Authors:** Federica Pessagno, Aliya Nur Hasanah, Panagiotis Manesiotis

**Affiliations:** School of Chemistry and Chemical Engineering, Queen's University Belfast David Keir Building, Stranmillis Road BT9 5AG Belfast Northern Ireland UK p.manesiotis@qub.ac.uk; Pharmaceutical Analysis and Medicinal Chemistry Department, Faculty of Pharmacy, Universitas Padjadjaran Jl Raya Bandung Sumedang KM 21,5 Jatinangor Indonesia

## Abstract

A novel approach towards recognition of sulfonylureas based on a polymerisable ion pair is presented. A solution association constant >10^5^ M^−1^ between the model target glibenclamide and 4-vinylbenzyltrimethylammonium methacrylate is measured, and the formation of 1 : 1 complexes verified. Subsequently prepared stoichiometrically imprinted polymers exhibit exceptionally high affinity and binding capacity for glibenclamide, owing to synergistic binding of both the neutral and deprotonated form of the drug by the ion pair monomer. The polymers are applied to the selective extraction of glibenclamide from blood serum samples, achieving recoveries of up to 98% and demonstrating excellent long-term stability, negating the need for regular sorbent regeneration.

## Abstract 2

Polymerisable ion pair captures both neutral and anionic form of acidic sulfonylurea drug in stoichiometrically imprinted polymer.

## Graphical abstract

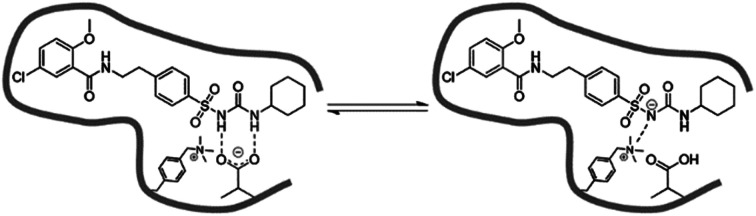

## Introduction

Sulfonylureas are a family of organic compounds with applications in medicine, mainly in the treatment of diabetes mellitus (type II), and agriculture, as herbicides. In both cases, such compounds are found in complex matrices and often at low concentrations, which makes their analytical determination a lengthy and laborious process, usually involving liquid–liquid or solid phase extraction, prior to analysis by HPLC-UV, HPLC-MS or capillary electrophoresis.^1–4^ Molecularly Imprinted Polymers (MIPs) have been previously used for the selective capture of glibenclamide (GLIB), a sulfonylurea drug. Wu *et al.* prepared MIP-coated micro-stir bars for the extraction of the sulfonylurea from herbal dietary supplements with recoveries of 81.9–101.4%,^5^ while Wang *et al.* achieved recoveries of 81.5–93.5% from health foods using dendritic grafting of MIPs onto magnetic nanoparticles.^6^ More recently, Ostovan *et al.* prepared hollow MIP nanoparticles for extraction of glibenclamide from urine with recoveries of 89.5%.^7^ In all these cases methacrylic acid was used as the functional monomer for recognition of the sulfonylurea.

**Fig. 1:**
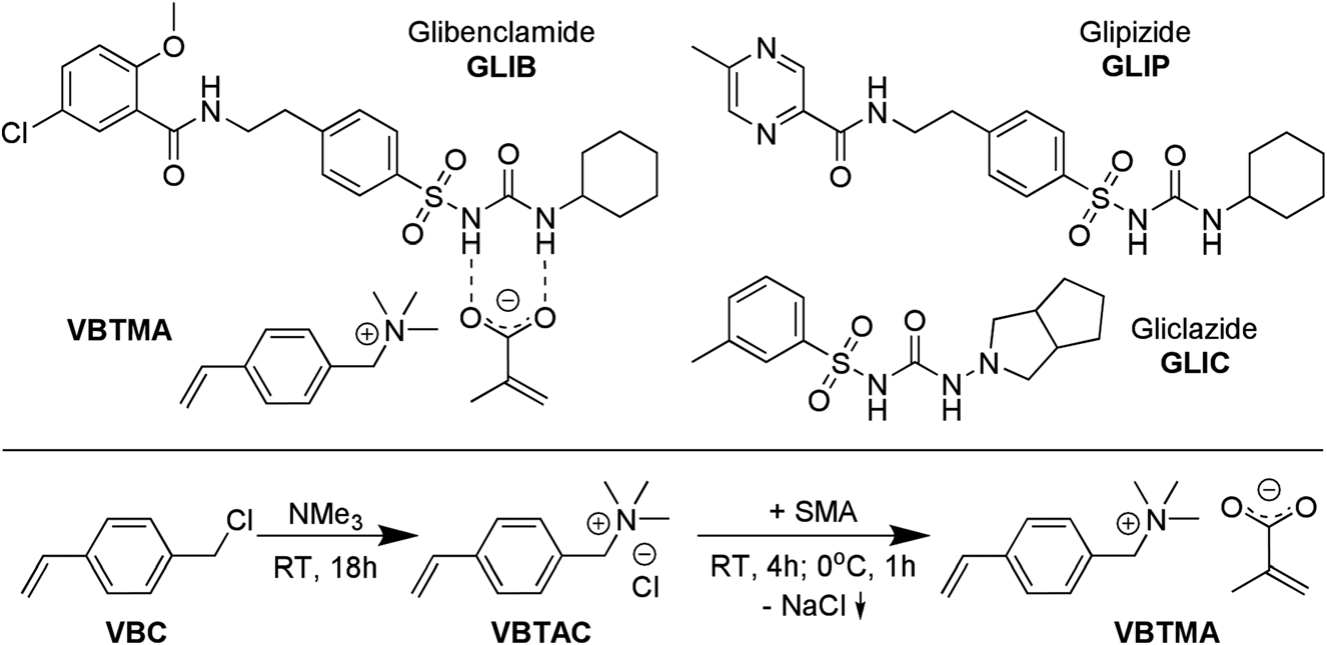
Chemical structures of VBTMA ion pair monomer, showing proposed primary interaction with GLIB, and structures of analogous sulfonylurea substances (top), and synthesis of VBTMA (bottom).

We have previously reported on our study of the interaction of glibenclamide (Fig. 1) with neutral and anionic receptors, and introduced tetrabutylammonium methacrylate (TBAM) as a novel recognition element for use in molecular imprinting,^8^ reversing the previously established polymerisable urea-carboxylate motif, studied by our group and others.^9–11^ We demonstrated that not only was the methacrylate anion capable of very strong association with the sulfonylurea moiety in solution, but under certain conditions it can deprotonate the acidic NH adjacent to the sulfonyl group, resulting in the formation of ‘narcissistic’ dimers^12^ between the neutral and anionic forms of GLIB, stabilised by the associated tetrabutylammonium cation. Consequently, when TBAM was used in the stoichiometric molecular imprinting of GLIB, the resulting polymers outperformed polymers prepared using acrylamide or methacrylic acid as the functional monomers. However, it was found that these polymers were ‘deactivated’ upon GLIB binding, by transfer of a proton from the template to the methacrylate moieties residing within the binding sites, thus negating the functional group complementarity between the two counterparts. This limitation was overcome by addition of a polymer regeneration step after each extraction cycle, using a dilute tetrabutylammonium hydroxide solution. Nonetheless, while no adverse effects on the stability or performance of the polymers were observed, it was decided to investigate alternative, more robust binding motifs.

Here, we wish to report, for the first time, the development of a novel polymerisable ion pair, whereby both anionic and cationic counterparts are permanently incorporated in the polymer matrix, and its application in the molecular imprinting of sulfonylureas. This approach is complementary to the field of ion-pair receptors, expertly reviewed in literature,^13^ as instead of employing a single receptor with heterotopic binding sites for both co-existing cationic and anionic partners of an ion pair, we employ a polymerisable ion pair to recognise both the neutral and anionic form of a sulfonylurea that do not co-exist but are different forms of the same molecule. Thus, even if the target molecule switches between the two forms by a change in the chemical environment, *e.g.* pH, the new polymer-bound receptor will be able to capture it, maximising the efficiency and application range of the material and revealing the true potential of the imprinted material. Furthermore, in a step change compared to previous reports of mixed ionic polymers^14^ and poly(ionic liquids) as molecular recognition elements,^15–18^ where one of the counterparts is mobile and can be exchanged during application of the material,^19^ the present design yields robust imprinted polymers that can be repeatedly used without loss in performance due to ion exchange, and without the need for regular regeneration. Furthermore, the novel materials exhibit exceptionally high affinity, as well as enhanced binding capacity and selectivity for the model sulfonylurea template, vastly outperforming previously reported sorbents.

## Experimental

### Materials and methods

Glibenclamide (GLIB), sodium methacrylate (SMA), 4-vinylbenzyl chloride (VBC), tetrabutylammonium hydroxide (TBAOH), tetrabutylammonium chloride (TBACl), ethyleneglycol dimethacrylate (EGDMA), 1-hydroxycyclohexyl phenyl ketone (UV initiator), acetic acid, trifluoroacetic acid (TFA), trimethylamine (TMA, 4.2 mol L^−1^ solution in ethanol), triethylamine (TEA), HPLC grade solvents, deuterated solvents, empty polypropylene solid-phase extraction (SPE) cartridges (3 mL) and 20 μm porous polyethylene frits were purchased from Sigma Aldrich (Gillingham, UK). Polymerisation inhibitors were removed from all monomers by filtration through a basic alumina column. Lithium bis(trifluoromethyl-sulfonyl)imide (LiNTf_2_) was purchased from Alfa Aesar (Heysham, UK). Glipizide (GLIP) was purchased from the Indonesian National Agency of Drug and Food Control. Gliclazide (GLIC) was provided by Dexa Medica Pharmaceuticals Industry (Tangerang, Indonesia). NMR spectra were collected on a Bruker Avance 600 MHz NMR spectrometer, and ^1^H NMR titrations and Job plots on a Bruker ECX 400 MHz NMR spectrometer (Coventry, UK). FT-IR spectra were recorded on a Perkin Elmer Spectrum 100 FT-IR spectrometer equipped with an ATR attachment (Seer Green, UK). An Agilent 1100 HPLC instrument equipped with photodiode array detector was used in for all chromatographic separations. Analyses were performed by isocratic elution using a 40 : 60 water/acetonitrile mixture containing 0.01% TFA as the mobile phase and a Phenomenex Kinetex™ C18 column (5 μm, 150 mm × 4.6 mm i.d.) (Macclesfield, UK). The flow rate was 1 mL min^−1^ and the detection wavelength was set at 230 nm. A 12-port Phenomenex vacuum manifold was used for SPE experiments (Macclesfield, UK). Blood samples were provided by the Indonesian Red Cross.

### Synthesis of 4-vinylbenzyltrimethylammonium methacrylate

4-vinylbenzyltrimethylammonium methacrylate (VBTMA) was prepared in two steps as follows: 1.53 g of inhibitor free VBC (10 mmol) and 20 mL of ethanol were transferred to a round-bottom flask and 4.75 mL (20 mmol) of a 4.2 M solution of TMA in ethanol were added. The reaction was allowed to proceed at room temperature for 18 h and the solvent was subsequently evaporated under reduced pressure. The product, 4-vinylbenzyltrimethylammonium chloride (VBTAC), was obtained as a white solid in quantitative yield. ^1^H NMR (600 MHz, DMSO-d_6_) *δ* 7.67–7.47 (m, 4H), 6.81 (dd, *J* = 17.7, 11.0 Hz, 1H), 5.96 (dd, *J* = 17.7, 0.8 Hz, 1H), 5.39 (dd, *J* = 10.9, 0.8 Hz, 1H), 4.56 (s, 2H), 3.35 (s, 9H); ^13^C NMR (151 MHz, DMSO-d_6_) *δ* 139.34, 136.32, 133.58, 128.29, 126.99, 116.68, 67.86, 52.17. HRMS: C_12_H_18_N^+^ calculated 176.1434, found 176.1358.

In order to obtain the final monomer, 2.12 g (10 mmol) of VBTAC were dissolved in 50 mL of ethanol and 1.08 g (10 mmol) of sodium methacrylate were added. The mixture was stirred for 4 h at room temperature, then cooled at 0 °C for 1 h and finally centrifuged at 3000 rpm to remove the NaCl formed. The supernatant was evaporated to dryness under reduced pressure to yield the product, VBTMA, as a white solid with 88% yield. ^1^H NMR (600 MHz, DMSO-d_6_) *δ* 7.68–7.46 (m, 4H), 6.81 (dd, *J* = 17.7, 11.0 Hz, 1H), 5.96 (dd, *J* = 17.7, 0.8 Hz, 1H), 5.52 (d, *J* = 3.4 Hz, 1H), 5.39 (dd, *J* = 10.9, 0.8 Hz, 1H), 4.99–4.89 (m, 1H), 4.56 (s, 2H), 3.05 (s, 9H), 1.76 (s, 3H); ^13^C NMR (151 MHz, DMSO-d_6_) *δ* 171.45, 146.24, 139.32, 136.33, 133.59, 128.33, 126.98, 116.66, 116.44, 67.86, 52.16, 20.88. A melting point of 160 °C was measured, however, the compound polymerised immediately upon melting.

### Synthesis of glibenclamide tetrabutylammonium salt

The tetrabutylammonium salt of GLIB (GLIB-TBA) was prepared by mixing of equimolar amounts of GLIB and TBAOH in methanol, followed by evaporation of the solvent under reduced pressure to yield GLIB-TBA as a white solid in quantitative yield. ^1^H NMR (600 MHz, DMSO-d_6_) *δ* 8.23 (s, br, 1H), 7.69 (dd, *J* = 2.8, 1.8 Hz, 1H), 7.67–7.63 (m, 2H), 7.50 (dd, *J* = 8.9, 2.8 Hz, 1H), 7.21 (d, *J* = 8.3 Hz, 2H), 7.15 (d, *J* = 8.9 Hz, 1H), 5.54 (s, 1H), 3.80 (s, 3H), 3.51 (t, *J* = 7.2 Hz, 2H), 3.20–3.12 (m, 8H), 2.83 (t, *J* = 7.2 Hz, 2H), 1.63–1.52 (m, 8H), 1.36–1.25 (m, 8H), 0.94 (t, *J* = 7.4 Hz, 12H); ^13^C NMR (151 MHz, DMSO-d_6_) *δ* 163.89, 156.24, 146.79, 140.53, 131.99, 130.05, 128.08, 126.99, 125.16, 124.78, 114.64, 58.00, 56.75, 49.00, 41.07, 40.54, 35.10, 33.95, 25.96, 25.28, 23.54, 19.68, 13.96.

### Synthesis of 4-vinylbenzyltrimethylammonium bis(trifluoro-methylsulfonyl) imide

4-vinylbenzyltrimethylammonium bis(trifluoromethylsulfonyl) imide (VBTANTf_2_) was prepared by addition of two-fold excess of LiNTf_2_ to an aqueous solution of 4-vinylbenzyltrimethyl ammonium chloride (VBTAC), followed by solvent extraction of the aqueous phase with chloroform, drying of the organic layer with MgSO_4_, and solvent evaporation under reduced pressure, to finally yield a white solid. ^1^H NMR (600 MHz, CDCl_3_) *δ* 7.44 (dd, *J* = 41.3, 8.2 Hz, 4H), 6.71 (dd, *J* = 17.6, 10.9 Hz, 1H), 5.83 (d, *J* = 17.6 Hz, 1H), 5.38 (d, *J* = 11.1 Hz, 1H), 4.41 (s, 2H), 3.08 (s, 9H); ^13^C NMR (151 MHz, CDCl_3_) *δ* 139.44 (s), 134.38 (s), 131.90 (s), 126.09 (s), 124.67 (s), 121.98–115.60 (q), 115.64 (s), 68.84 (s), 51.78–51.50 (t); ^19^F NMR (565 MHz, CDCl_3_) *δ* −78.94 (s).

### ^1^H NMR titration experiments

The solution interactions of GLIB and GLIB-TBA with VBTMA, VBTAC and VBTANTf_2_, as well as the complexation of GLIB with TBACl, were studied by ^1^H NMR titrations in DMSO-d_6_. Thus, to a 1.0 mmol L^−1^ solution of the host (GLIB or GLIB-TBA), increasing amounts of each guest were added, until at least a 10-fold excess was reached. The complexation-induced shift (CIS) of several protons was followed and titration isotherms were constructed. The stoichiometry of the selected monomer–template complexes was confirmed using Job's method of continuous variation. Hence, equimolar solutions (10.0 mmol L^−1^) of the host and each guest were mixed in different ratios and a plot of Δ*δ* against the molar fraction of monomer multiplied by the CIS (*X*_*i*_ × Δ*δ*) was constructed.

### Preparation of imprinted polymers

Stoichiometrically imprinted and corresponding non-imprinted polymers, P_GLIB_, P_GLIBTBA_ and NP respectively, were prepared by photochemically initiated free radical polymerisation. The compositions of all prepared polymers are presented in Table 2. Briefly, the template and the selected functional monomer were transferred into to a glass vial and mixed with the porogen. Upon complete dissolution, the cross-linker was added followed by the initiator. The resulting pre-polymerisation solutions were degassed by ultra-sonication for 5 min, purged with argon and then hermetically sealed. The vials were then placed in the chamber of a UVP CX-2000 UV curing reactor (UVP, Jena, Germany) and irradiated at 360 nm for 3 hours at room temperature. The resulting rigid monoliths were coarsely ground and washed with methanol in a Soxhlet apparatus for 24 h, in order to remove the template and any unreacted monomers. The coarse polymer particles were further ground using a mortar and pestle, wet-sieved with acetone, and the 25–50 μm fraction was collected, dried and stored at room temperature. The corresponding non-imprinted polymers were prepared in a similar fashion, omitting addition of the template to the pre-polymerisation mixture.

**Table 1:** Apparent association constants (*K*_a_, M^−1^) measured by ^1^H NMR titration experiments in DMSO-d_6_

Guest	Host	
	GLIB	GLIB-TBA
VBTMA	>10^5^	53 ± 6
VBTAC	34 ± 5	No binding
VBTANTf_2_	No binding	22 ± 3
TBACl	3452 ± 230	No binding

**Table 2:** Compositions of the polymers reported in this study

Polymer ID	Functional monomer	Template	Cross-linker	Porogen
P_GLIB_	VBTMA	GLIB	EDMA	CHCl_3_
NP	VBTMA	—	EDMA	CHCl_3_
P_GLIB2_	TBAM/MAA 1 : 1	GLIB	EDMA	CHCl_3_
NP_2_	TBAM/MAA 1 : 1	—	EDMA	CHCl_3_
P_GLIBTBA_	VBTAC	GLIB-TBA	EDMA	DMSO
P_GLIBTBA(NTf2)_	VBTANTf_2_ (exchanged)	GLIB-TBA	EDMA	DMSO
P_GLIBTBA(XL)_	—	GLIB-TBA	EDMA	DMSO/CHCl_3_

P_GLIBTBA(NTf2)_ was prepared from P_GLIBTBA_ by exchange of chloride counter anions with bis-triflimide. Briefly, 0.5 g of P_GLIBTBA_ were suspended in 10 mL of distilled water containing 0.5 g of LiNTf_2_. The suspension was stirred at room temperature overnight and then polymer particles were filtered, washed with distilled water and dried under reduced pressure prior to use. Polymers P_GLIBTBA2_ and NP_2_ were prepared as described in our previous publication.^8^ An additional control polymer, P_GLIBTBA(XL)_, was prepared in a similar fashion to P_GLIBTBA_, but without the addition of a functional monomer.

### Rebinding experiments

Polymer affinity and capacity for each analyte were measured using equilibrium rebinding experiments performed in acetonitrile. Thus, 10 mg of each polymer were transferred in 2 mL glass vials and incubated with 1.5 mL of analyte solution of increasing concentrations (0.0–3.0 mmol L^−1^) for 24 hours. The supernatants were then analysed by HPLC using the method described above. The amount of analyte bound to the polymer was calculated by subtracting the amount determined after the rebinding experiment from the starting amount of the drug. The results were plotted as concentration of free analyte in solution (mol L^−1^) *vs.* the amount of analyte bound on the polymers (μmol g^−1^) to produce binding isotherms that were fitted using the appropriate binding model.

### Solid phase extractions

50 mg of imprinted or non-imprinted polymer particles (25–50 μm) were dry packed in 3 mL SPE cartridges using 20 μm porous polyethylene frits. Blood serum samples were prepared by centrifugation of the collected blood at 8000 rpm for 5 minutes at 14 °C and careful collection of the clear top layer. Blood serum samples were spiked with 5 mg L^−1^ of GLIB in 5% acetonitrile in water. Following an extensive optimisation process, the final extraction protocol consisted of an initial conditioning step with 1 mL of 5% acetonitrile in water, loading 2 mL of the spiked blood sample, followed by an aqueous wash (1 mL), a wash with 2 mL of 0.01% TEA in CHCl_3_, and a final elution with 0.5 mL of 1% acetic acid in methanol. Full vacuum was applied to the cartridges between each step for 2 minutes. In order to test the specificity of the prepared polymers, an equimolar mixture of GLIB, GLIC and GLIP (5 mg L^−1^ each) in 5% acetonitrile in water was spiked into blood serum samples and applied onto the SPE cartridges. The collected fractions were analysed by HPLC using the method described above.

## Results and discussion

### Host-guest interactions in solution

**Fig. 2:**
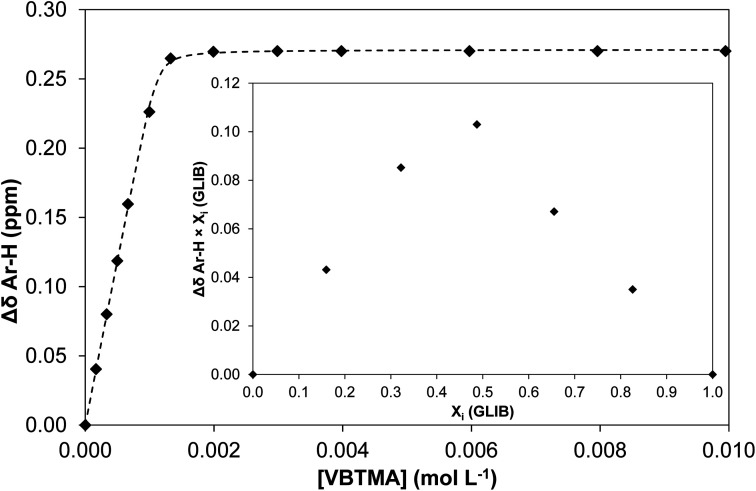
Binding isotherm obtained during ^1^H NMR titration of GLIB with VBTMA in DMSO-d_6_. Inset: Job plot for the association of GLIB with VBTMA in DMSO-d_6_, where the formation of 1 : 1 complexes is verified.

**Fig. 3:**
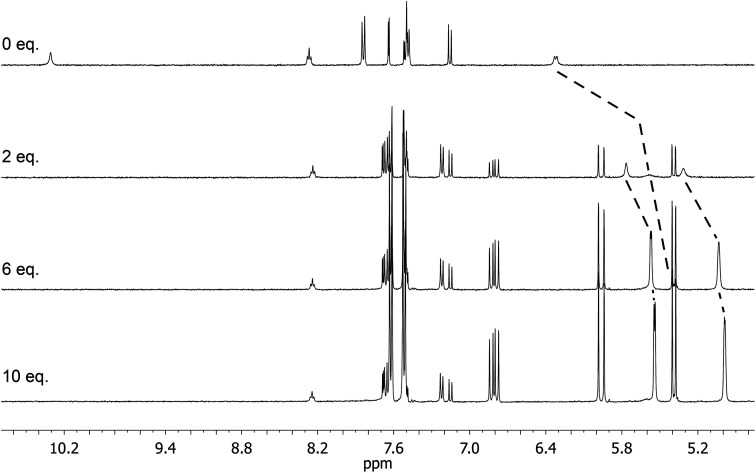
Overlay of characteristic ^1^H NMR spectra (DMSO-d_6_) obtained during the titration of GLIB *vs.* VBTMA, showing the disappearance of the acidic sulfonylurea proton and the movement of the second sulfonylurea proton, as well as the movement of the methacrylate vinyl protons. From top to bottom, 0 eq., 2 eq., 6 eq. and 10 eq. of VBTMA added to GLIB.

**Fig. 4:**
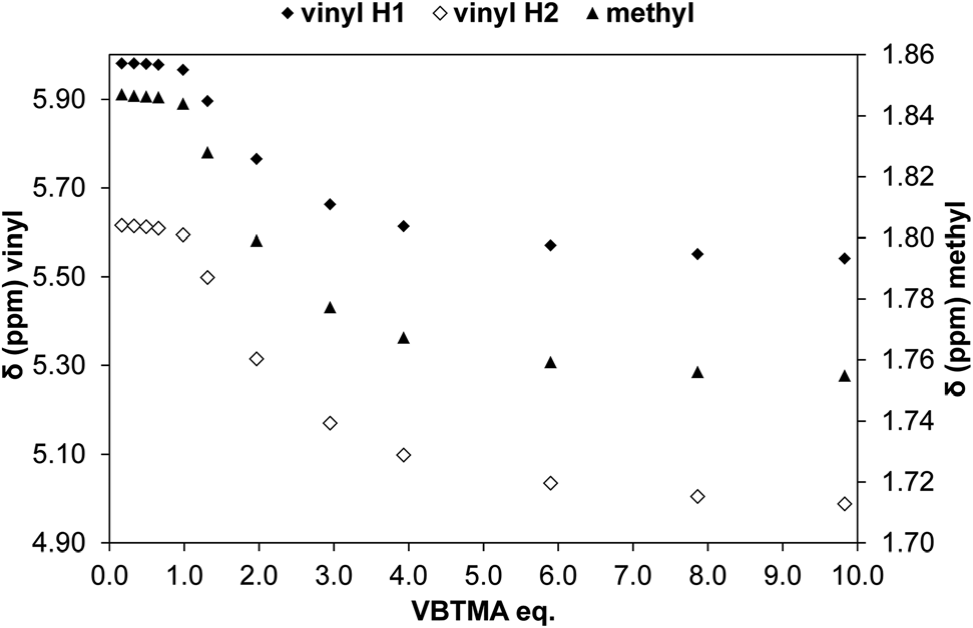
Change in chemical shift of the two vinyl protons (diamonds, left axis) and methyl protons (triangles, right axis) of methacrylate during the titration of GLIB *vs.* VBTMA in DMSO-d_6_.

The solution association of GLIB with the novel polymerisable ion pair system VBTMA was studied by a series of ^1^H NMR titrations in DMSO-d_6_. Several additional equilibria were also investigated in order to explain the behaviour observed by subsequently prepared imprinted polymers. The obtained association constants are outlined in Table 1. As seen in Fig. 2, VBTMA interacts strongly with GLIB, forming 1 : 1 complexes with an estimated stability constant *K*_a_ > 10^5^ M^−1^, too strong to accurately determine by ^1^H NMR titration. Further insights into the nature of the formed complexes were offered by closer inspection of the collected ^1^H NMR spectra (Fig. 3), in particular the chemical shifts of the methacrylate protons and the methyl and methylene protons of the counter-cation. Thus, during the early stages of the titration experiment, where GLIB (host) was in excess compared to VBTMA (guest), and up to a ratio GLIB : VBTMA of 1 : 1, the signals corresponding to the two methacrylate protons were poorly defined and showed almost no change (Fig. 4). The chemical shift of those protons was also down-field from their position in the spectrum of the free monomer. Furthermore, the disappearance of the signal corresponding to the acidic sulfonylurea NH (initially at 10.31 ppm), and the concurrent up-field shift of the second sulfonylurea NH (initially at 6.32 ppm), are consistent with deprotonation of GLIB and protonation of methacrylate. Once an excess of VBTMA was added, the signals attributed to the methacrylate group moved towards their corresponding positions in the spectrum of the free monomer (4.99 ppm and 5.54 ppm), while the second sulfonylurea proton nearly disappeared under the methacrylate signal (5.59 ppm). The deprotonation event was also evidenced by the movements of the signals corresponding to the aromatic protons adjacent to the sulfonylurea group, whereby up-field shifts from 7.84 ppm to 7.86 ppm, and 7.48 ppm to 7.22 ppm were observed. It is noteworthy that the positions of the signals corresponding to GLIB protons at the end of the titration, are identical to the signals of GLIB-TBA, which further supports the deprotonation mechanism. Observation of the peaks corresponding to the methyl and methylene groups of the positively charged counterpart, show a gradual down-field movement of the signals throughout the titration, however, upon closer inspection of the plotted curves, the chemical shift change is sharper up to the 1 : 1 point of the experiment and becomes shallower when excess VBTMA has been added (see ESI†). This behaviour hints at an interaction of the quaternary ammonium cation with GLIB^−^ that is possibly stronger that the force between the two partners of the polymerisable ion pair. This is a crucial attribute of the ion pair monomer as it means that when exposed to the sulfonylurea, both counterparts will favourably bind to the neutral or deprotonated form of the latter, and the interaction between them will not hinder the association to the third party. In an attempt to quantify this interaction, a titration experiment between GLIB-TBA and VBTMA revealed a weak yet significant association (*K*_a_ = 53 M^−1^) of the deprotonated host with the quaternary ammonium functionality of the ion pair monomer. Interestingly, movement was only detected for the signals corresponding to either the methyl or the methylene groups of VBTMA, and not the methacrylate protons.

The binding mechanism was further investigated by titration of GLIB and GLIB-TBA *vs.* VBTAC. In the former case, weak association, again accompanied by movement of the methyl and methylene peaks of VBTAC, as well as the two sulfonylurea protons, was observed, which was attributed to interaction of the chloride ion with the sulfonylurea (see ESI†). This was supported by the strong association of GLIB with TBACl (*K*_a_ = 3452 M^−1^). The bulky tetrabutylammonium cation associates weakly with chloride, thus not hindering the interaction of the halide anion with the sulfonylurea. No binding was observed between GLIB-TBA and either VBTAC or TBACl, due to the electrostatic repulsion between the negative charged sulfonylurea and chloride anions. Lastly, when chloride was exchanged with the larger, non-coordinating, bis-triflimide anion, weak binding with the positive quaternary ammonium monomer was observed (*K*_a_ = 22 M^−1^), while no interaction with GLIB could be detected. It was thus concluded that the binding between GLIB and VBTAC is mediated by the formation of chloride bridges, while the GLIB⋯Cl^−^ complexes electrostatically repel GLIB-TBA.

### Evaluation of polymer performance

Physico-chemical characterisation of the prepared materials was conducted by surface area analysis and FT-IR. The specific surface area of P_GLIB_ was 67.2 m^2^ g^−1^ with a pore diameter of 23.1 Å, and the corresponding values for NP were 73.1 m^2^ g^−1^ and 37.5 Å. Both sets of values are in the same range, so the porous structure of the two polymers should not influence the results of the subsequent rebinding experiments. FT-IR analysis revealed all the characteristic peaks for the incorporated functional groups (carboxylate: 1574 cm^−1^, 1388 cm^−1^; C=O (ester): 1722 cm^−1^; methylene bend: 1451 cm^−1^; C–C skeletal stretch: 1138 cm^−1^), while the spectra of both polymers were nearly identical, suggesting that the presence of the template did not impact the progress of the polymerisation reaction or the relative reactivity of the ion pair and cross-linking monomer, and that it had been fully removed during the polymer washing procedure (see ESI†).

**Fig. 5:**
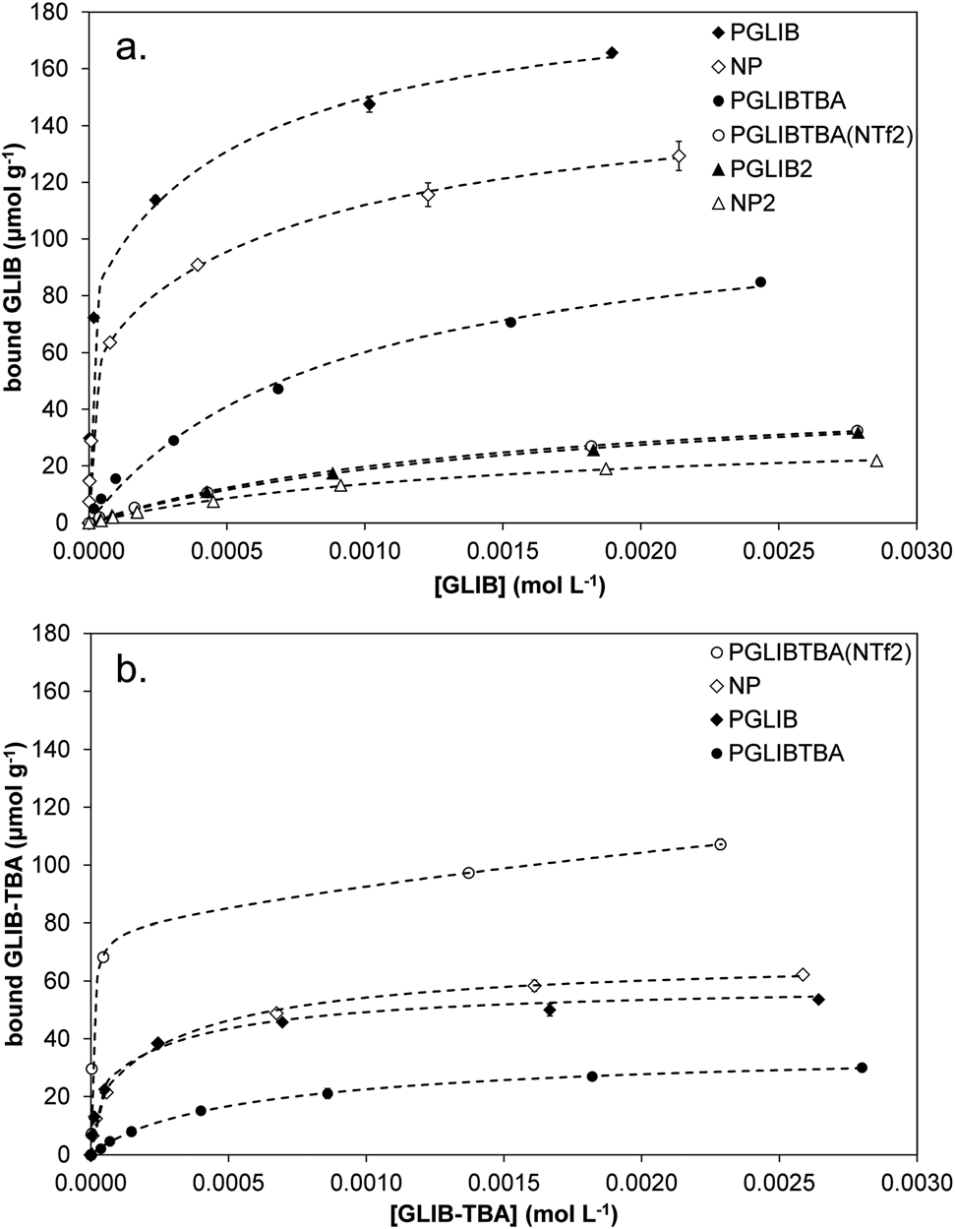
Equilibrium rebinding isotherms of (a) GLIB and (b) GLIB-TBA on the prepared polymers.

Evaluation of the polymer binding performance was conducted by means of equilibrium rebinding experiments, whereby binding isotherms were constructed as shown in Fig. 5a. The derived fitting parameters are presented in Table 3. Upon observation of the isotherms for the binding of GLIB on the corresponding imprinted and non-imprinted polymers, exceptionally strong binding is evident at the low concentration range, and virtually all of the template is removed from the supernatant by both polymers up to the concentration of 0.5 mmol L^−1^, following which point the isotherms level off rapidly, indicating that the saturation point has been reached. Furthermore, P_GLIB_ greatly outperformed the previously reported P_GLIB2_ by nearly two orders of magnitude in affinity and over three-fold in binding capacity. Interestingly, the binding isotherms for GLIB on P_GLIB_ and NP could not be fitted to the Langmuir model, in contrast to their previous counterparts P_GLIB2_ and NP_2_, and a bi-Langmuir model was used instead. Although both models offer a simplified approximation of the type of binding sites present in imprinted polymers, this result suggests the presence of very high affinity sites, in which methacrylate and quaternary ammonium groups bind GLIB synergistically, and lower affinity sites, where functionality orientation is sub-optimal. The chloride bridge mediated binding mechanism between GLIB and the quaternary ammonium moiety was verified by rebinding of GLIB on P_GLIBTBA_ and P_GLIBTBA(NTf2)_, both polymers containing only the positively charged quaternary ammonium monomer, with either chloride or bis-triflimide as counter anions. Thus, although significantly weaker binding and lower capacity for GLIB was observed on P_GLIBTBA_ compared to P_GLIB_, when the chloride counterion of P_GLIBTBA_ was exchanged with bis-triflimide (P_GLIBTBA(NTf2)_), GLIB binding capacity was further reduced by nearly a factor of two.

**Table 3:** Affinity constants (*K*_a_, L mol ^−1^) and number of binding sites (*N*, μmol g^−1^) calculated using the Langmuir or bi-Langmuir binding model, from equilibrium rebinding experiments presented in Fig. 5

Polymer ID	GLIB		GLIB-TBA	
	*K*_a_ (L mol ^−1^)	*N* (μmol g^−1^)	*K*_a_ (L mol ^−1^)	*N* (μmol g^−1^)
P_GLIB_	1.7 ± 0.4 × 10^3^	108.8 ± 7.1	4.8 ± 1.2 × 10^3^	37.0 ± 3.9
	3.8 ± 0.6 × 10^5^	80.8 ± 4.9	1.2 ± 0.5 × 10^5^	18.2 ± 4.4
NP	1.2 ± 0.3 × 10^3^	93.7 ± 5.0	2.5 ± 0.6 × 10^3^	47.9 ± 3.8
	1.2 ± 0.2 × 10^5^	62.0 ± 3.6	6.3 ± 2.5 × 10^4^	20.4 ± 4.6
P_GLIB2_^a^	6.0 ± 0.5 × 10^2^	50.7 ± 2.3	No binding	
NP_2_^a^	7.0 ± 0.4 × 10^2^	33.1 ± 1.0	No binding	
P_GLIBTBA_	1.1 ± 0.2 × 10^3^	113.3 ± 6.9	1.9 ± 0.1 × 10^3^	35.1 ± 0.7
P_GLIBTBA(NTf2)_	6.5 ± 0.6 × 10^2^	50.2 ± 2.0	1.4 ± 0.2 × 10^2^	115.3 ± 12.3
			1.5 ± 0.8 × 10^5^	78.2 ± 7.1
P_GLIBTBA(XL)_	No binding		No binding	

a Previously published data.^8^

Following the rebinding experiments of GLIB-TBA (Fig. 5b), the deprotonated analogue of GLIB, it was found that the overall binding affinity and capacity of both P_GLIB_ and NP for GLIB-TBA was lower by a factor of three compared to GLIB, while imprinting selectivity was also lost. These results verify the proposition that the functional group responsible for selective binding of GLIB is methacrylate, as in its absence binding is diminished. Furthermore, the binding of GLIB-TBA on its corresponding imprinted polymer, P_GLIBTBA_, was significantly lower than on P_GLIB_, suggesting again that the presence of chloride results in repulsive forces between the interacting species. When exchanged with bis-triflimide, which does not hinder the interaction between the positively charged quaternary ammonium groups and the negatively charged deprotonated sulfonylurea, P_GLIBTBA(NTf2)_ exhibits a five-fold higher binding capacity for GLIB-TBA, further supporting the proposed binding mechanism. As additional proof of the importance of the functional monomer in the binding process, a polymer prepared without any functional monomer, P_GLIBTBA(XL)_, showed no binding for either the neutral or deprotonated form of the sulfonylurea drug.

It is noteworthy that VBTAC has been previously used in conjunction with MAA for the preparation of ion exchange MIPs used in the extraction of anionic sweetener acesulfame K from wastewater samples, however, in that case VBTAC acted as a phase transfer agent and no ion pair was formed with MAA, resulting in overall poor selectivity in the presence of other anionic compounds.^19^

### Solid phase extraction of blood serum samples

**Fig. 6:**
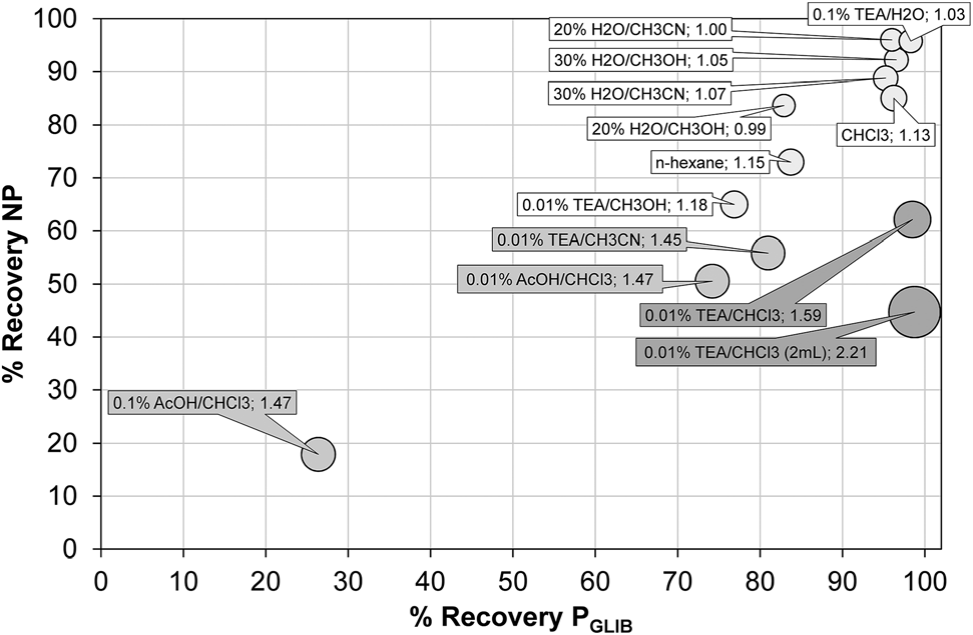
Optimisation of GLIB recovery using different SPE washing step conditions on P_GLIB_ and NP polymers. Size of each circle is proportional to the corresponding imprinting factor.

**Fig. 7:**
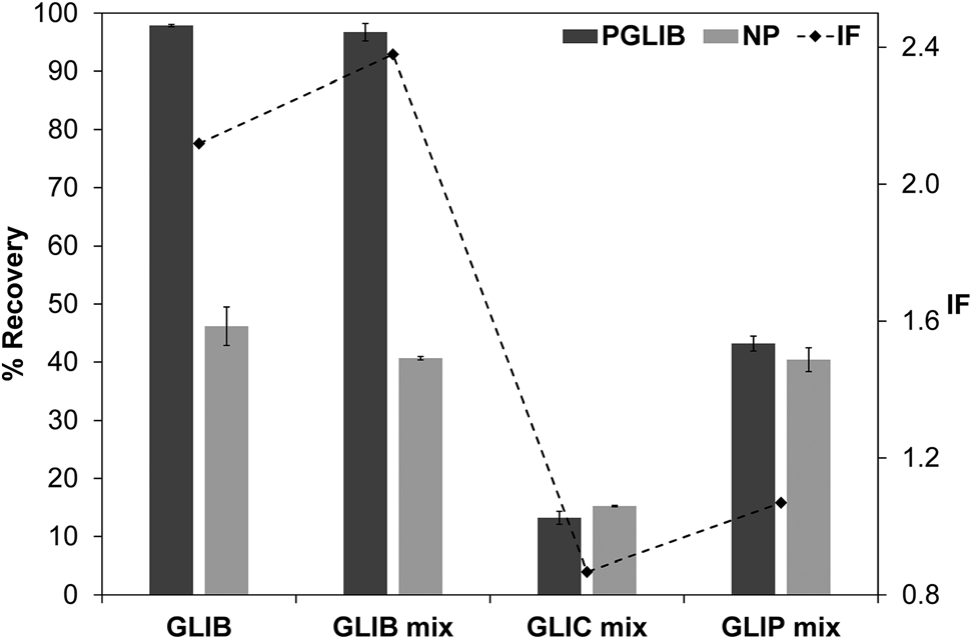
Recovery (%) of GLIB from spiked blood serum samples, compared to recovery of GLIB, GLIC and GLIP from equimolar spiked blood serum samples, using the optimised SPE procedure (bars – left axis), and corresponding imprinting factors for each analyte (diamonds – right axis).

The exceptionally strong binding for GLIB exhibited by the novel materials presented here, especially at lower concentrations, suggests these imprints could act as molecular “traps”, able to selectively recognise the sulfonylurea drug in complex matrices, such as blood serum. We thus opted to use solid phase extraction (SPE) as a simple and rapid tool for the development of an optimised extraction protocol. Loading of blood serum solutions spiked with 5 mg L^−1^ of the drug on P_GLIB_ and NP resulted in near quantitative capture of GLIB, suggesting the predominance of non-specific, hydrophobic interactions under these conditions. A systematic study of the so-called molecular recognition step was then conducted, whereby a wide range of solvent mixtures with acidic or basic modifiers were tested, aiming to find a solvent system that promotes specific interactions, without compromising the final recovery of the target. As seen in Fig. 6, mixtures of water with methanol, acetonitrile or triethylamine, resulted in final recoveries >80% from both polymers, however imprinting factors (IF), defined as the recovery of the drug on P_GLIB_ over the recovery on NP, ranged from 0.99 to 1.07. When more polar solvents, such as methanol and acetonitrile, modified with triethylamine, were used in the washing step, a marginal improvement in selectivity was observed (1.18 and 1.45 respectively), although recoveries on P_GLIB_ dropped below 80%, which was below the desirable level. Finally, chloroform was tested, as it is frequently hypothesised that using the polymerisation porogen as a washing step solvent will assist the polymer to recover the three-dimensional structure generated during the polymerisation. Thus, an IF of 1.13 was initially obtained, which increased to 1.47 when 0.01–0.1% acetic acid was added, but with a concomitant decrease in recovery to less than 30%. Addition of 0.01% TEA in chloroform resulted in IF of 1.59, which was further improved to a maximum value of 2.21 when the volume of the washing solution was increased to 2 mL. Under these optimised conditions, 98% of GLIB was recovered from blood serum using the imprinted polymer, and 46% using the non-imprinted polymer. The specificity of the prepared polymers was probed by analysis of blood serum samples spiked with equal concentrations of GLIB and two competing sulfonylureas, GLIC and GLIP, each at 5 mg L^−1^ (Fig. 7). Thus, it was shown that recoveries for GLIB on P_GLIB_ remained unaffected in the presence of the competing analytes, whose recoveries were 13% and 43% respectively. Calculated corresponding imprinting factors were 0.86 and 1.07 respectively, proving that the imprinting process had generated predominantly GLIB-specific binding sites and not generic sulfonylurea binding sites.

It should be noted that the above optimisation process of over 100 blood serum extraction cycles was conducted using a set of three cartridges for each polymer and that, in contrast to our previously reported sulfonylurea binding materials, no regeneration steps were required after each extraction cycle.

### Binding mechanism

**Fig. 8:**
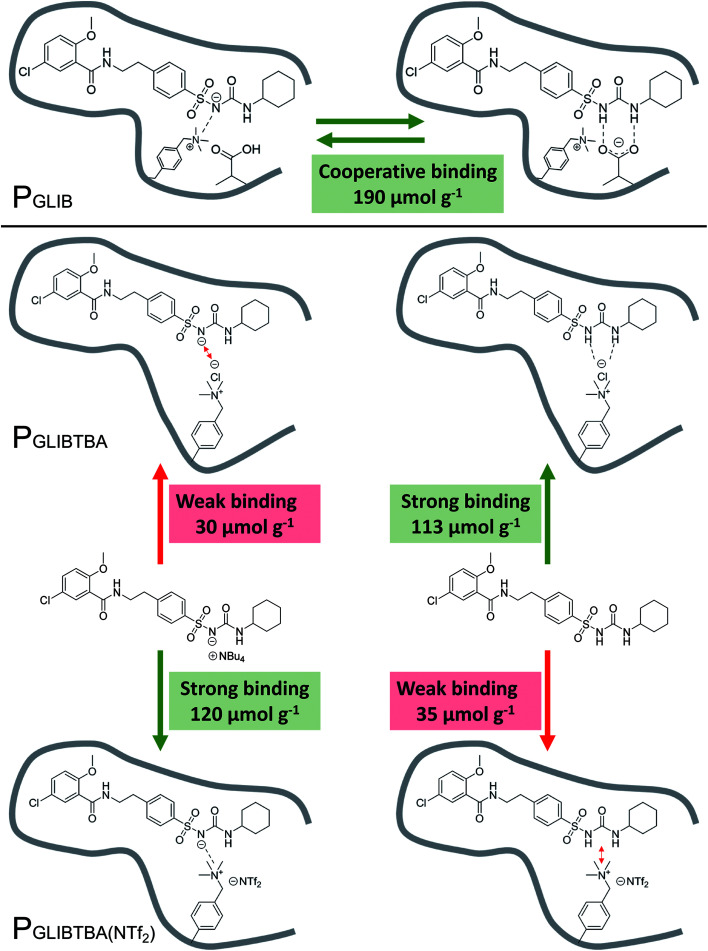
Overview of binding mechanism between the neutral or anionic forms of GLIB and P_GLIB_, P_GLIBTBA_ and P_GLIBTBA(NTf2)_, showing the corresponding binding capacities.

The binding mechanism between the novel polymerisable ion pair and the sulfonylurea drug, as elucidated by a series of solution interaction and equilibrium rebinding experiments, can be summarised as follows: using VBTAC as the sole functional monomer to imprint the deprotonated form of GLIB, results in weak template binding due to electrostatic repulsion between chloride and the deprotonated sulfonylurea, but stronger binding for neutral GLIB, *via* formation of chloride bridges (Fig. 8). Subsequent exchange of chloride with bis-triflimide “switches on” the binding sites for recognition of GLIB-TBA, but “switches off” the binding of neutral GLIB. These observations support the hypothesis that following initial binding of methacrylate to the sulfonylurea moiety, which has been shown previously to result in deprotonation of the acidic group and formation of GLIB anions, the latter is captured by the adjacent positively charged quaternary ammonium units.

This is the first report of a co-operative binding mechanism within an imprinted polymer, which is capable of binding both the neutral and dissociated form of a target substance, greatly enhancing the overall binding performance. It also offers direct evidence that the binding mechanism observed in solution by ^1^H NMR studies, still applies during the interaction of the drug with the imprinted polymer.

## Conclusions

A new concept of molecular imprinting, and its application to the recognition of a sulfonylurea antidiabetic drug, were demonstrated in this report. We have introduced, for the first time, a polymerisable ion pair as a binding element comprising a negatively charged methacrylate group and a positively charged quaternary ammonium counterion. The ion pair monomer exhibited exceptionally strong affinity for GLIB in solution and formed 1 : 1 complexes with the drug with *K*_a_ > 10^5^ M^−1^, while we have shown how the deprotonation of GLIB by methacrylate results in binding of GLIB anions by the positively charged partner of the ion pair. The new receptor motif was used in the preparation of stoichiometrically imprinted polymers for GLIB, which were capable of quantitative binding of the drug under static conditions up to concentrations of 0.5 mmol L^−1^ and total binding capacities improved by at least three-fold compared to previously reported materials.

With both counterparts of the ion pair monomer being permanently immobilised in the polymer matrix, we have overcome prior limitations imposed by the mobility of the counter-cation, which resulted in unstable materials that required regeneration after each application cycle. Indeed, we have shown that the new materials were capable of recoveries of GLIB from spiked blood serum up to 98% with imprinting factors of 2.21, while we were able to complete a study of over 100 blood serum extraction cycles using just three polymer cartridges, and without the need for intermediate regeneration steps, demonstrating the remarkable robustness of the new ion-pair based imprints. The approach presented here represents a paradigm shift in polymer based molecular recognition, and we are currently exploring the use of ion pair monomers in the recovery of sulfonylureas and related substances from a variety of complex matrices.

## Conflicts of interest

There are no conflicts to declare.

## Supplementary Material

RA-008-C8RA01135D-s001
