# Supplementary material for: Molecularly imprinted ‘traps’ for sulfonylureas prepared using polymerisable ion pairs
Source: RSC Adv. 2018 Apr 17;8(26):14212–20. doi: 10.1039/c8ra01135d (PMC9079912; doi:10.1039/c8ra01135d)
Supplement: RA-008-C8RA01135D-s001 [file RA-008-C8RA01135D-s001.pdf]

Electronic Supplementary Material (ESI) for RSC Advances.
This journal is © The Royal Society of Chemistry 2018

Supplementary Information

Molecularly imprinted 'traps' for sulfonylureas prepared using polymerisable ion pairs

Federica Pessagno[1], Aliya Nur Hasanah[2] and Panagiotis Manesiotis*[1]

Contents

Figures S1-S11: NMR spectra of all synthesised compounds.

Figure S12: ^{1}H NMR titration isotherm and Job plot of GLIB *vs.* TBACl.

Figure S13-15: Characteristic NMR titration spectra overlays

Figure S16. Change in chemical shift of the methyl groups and methylene protons of 4-vinylbenzyltrimethylammonium during the titration of GLIB *vs.* VBTMA.

Figure S17. GLIB recovery (%) on P_{GLIB} and NP, at each step of the optimised SPE protocol.

Figure S18. FT-IR spectra of P_{GLIB} and NP.

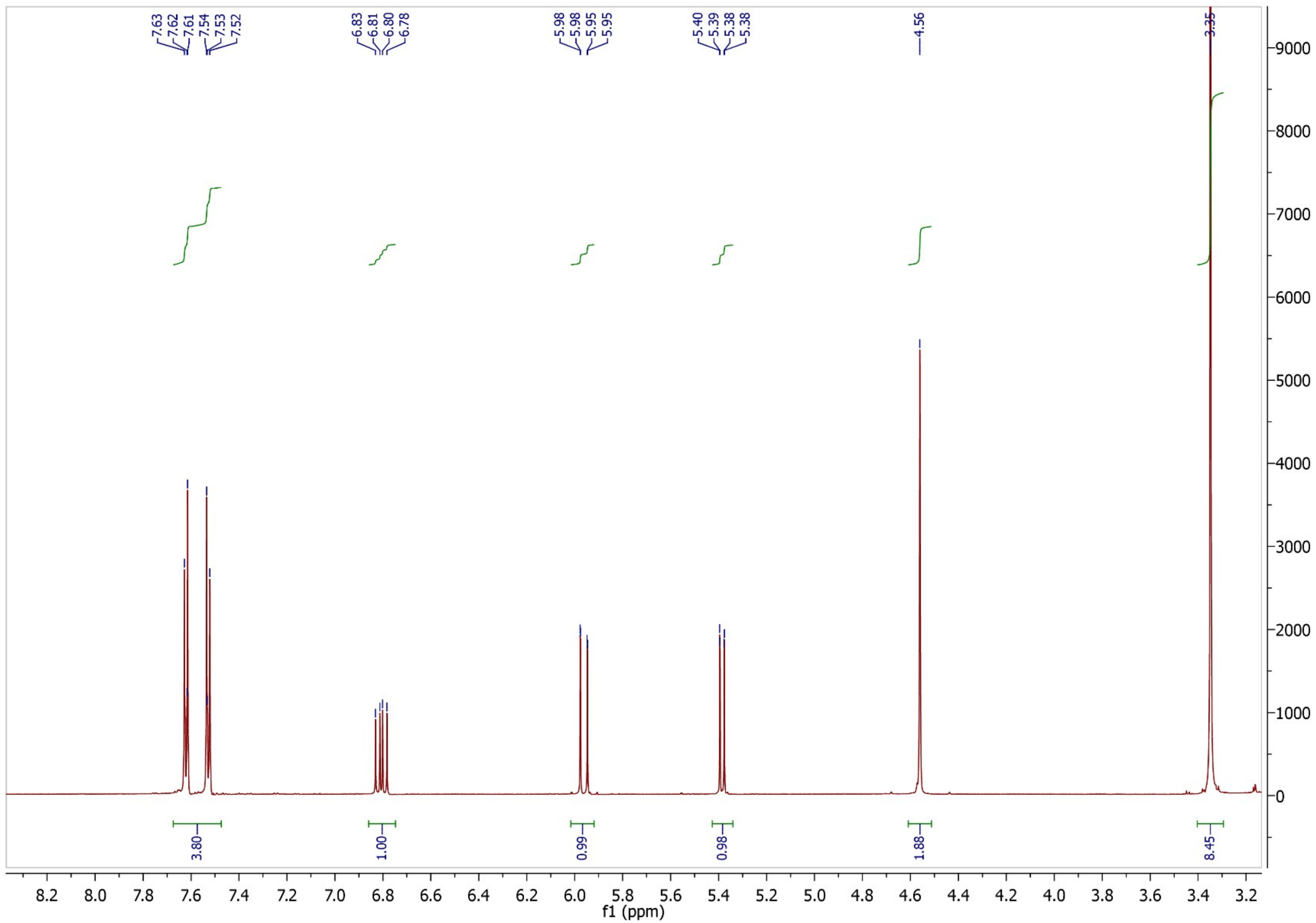

Figure S1. ^{1}H NMR spectrum of VBTAC (600MHz, DMSO).

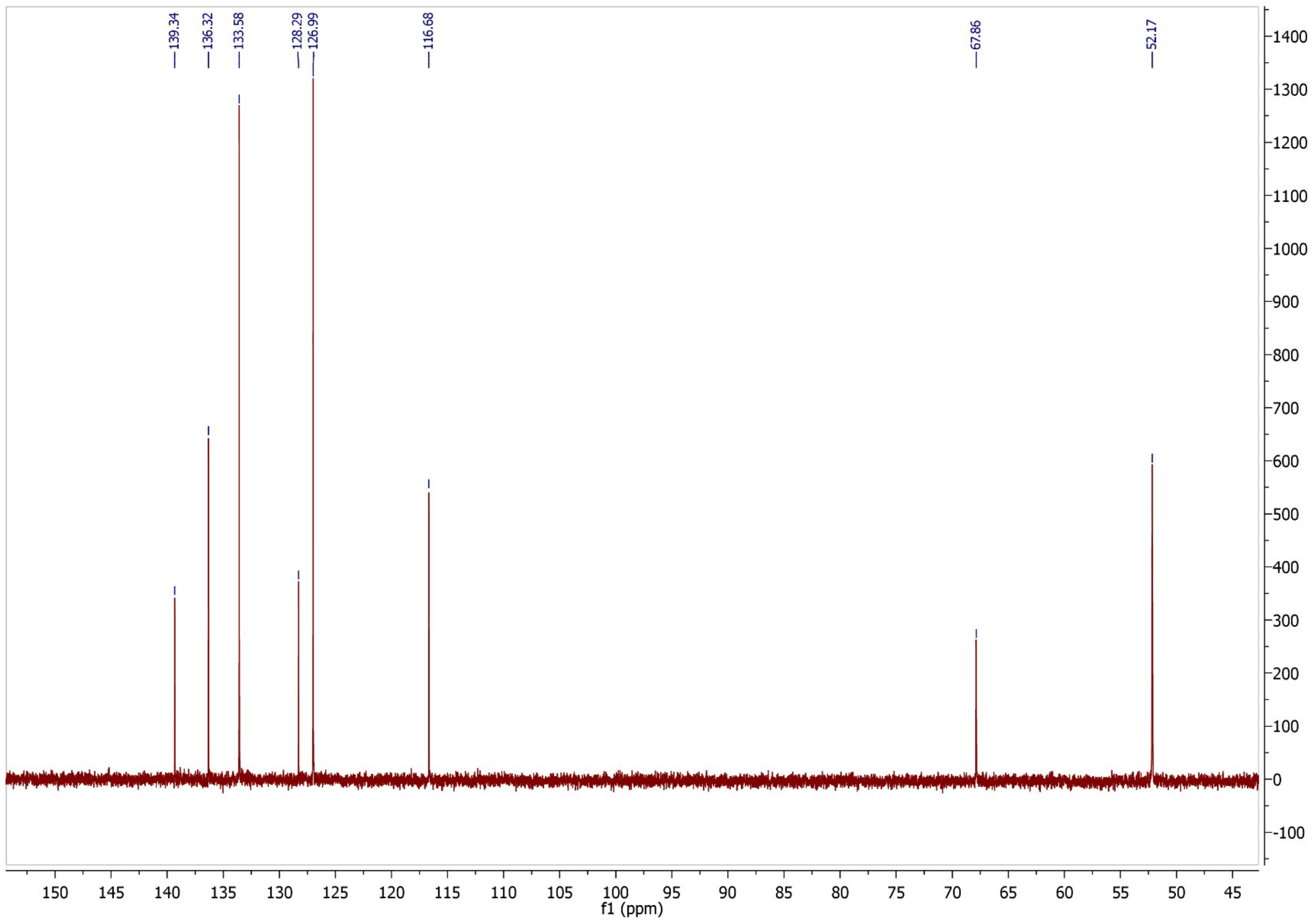

Figure S2. ^{13}C NMR spectrum of VBTAC (151MHz, DMSO).

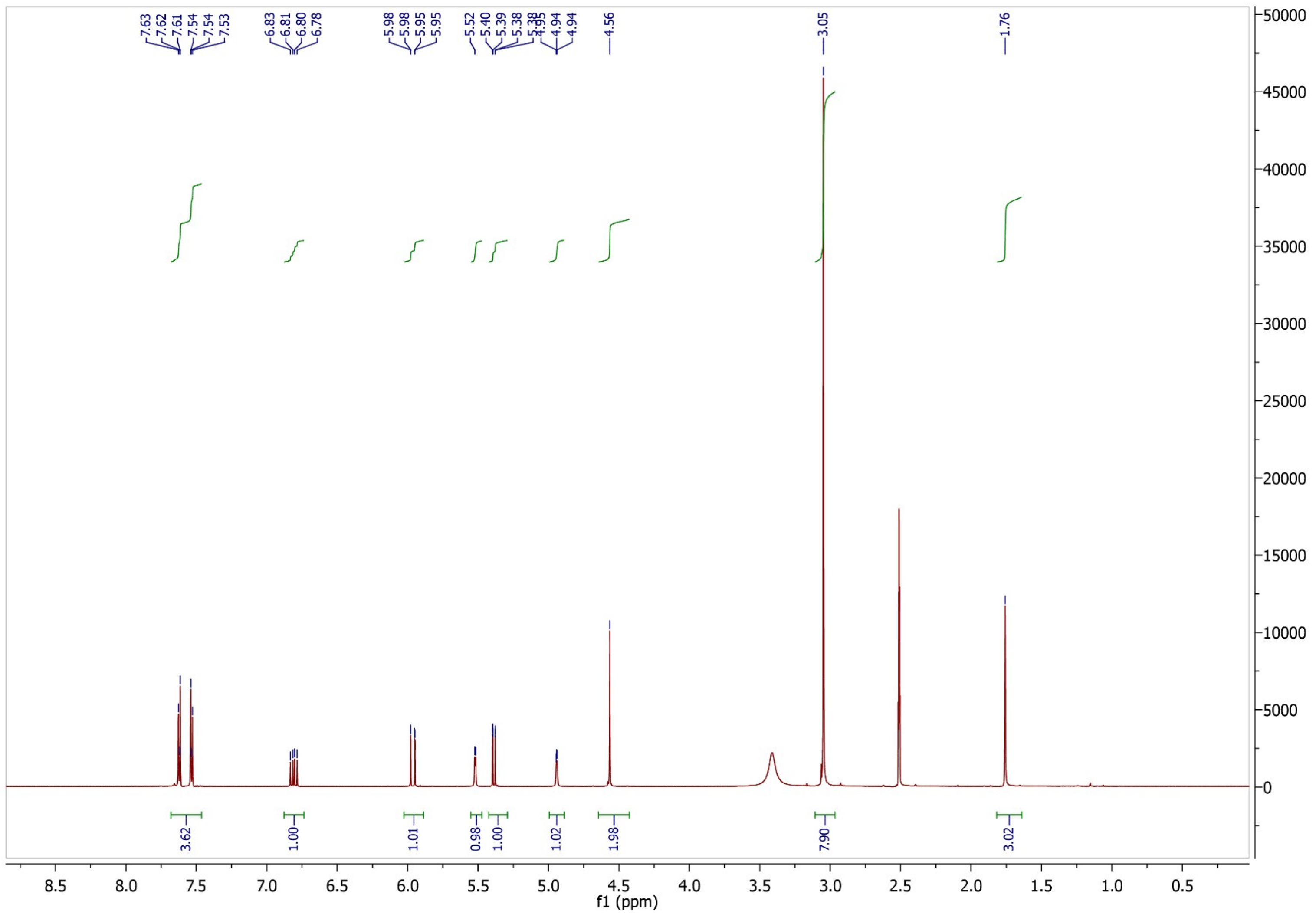

Figure S3. ^{1}H NMR spectrum of VBTMA (600MHz, DMSO).

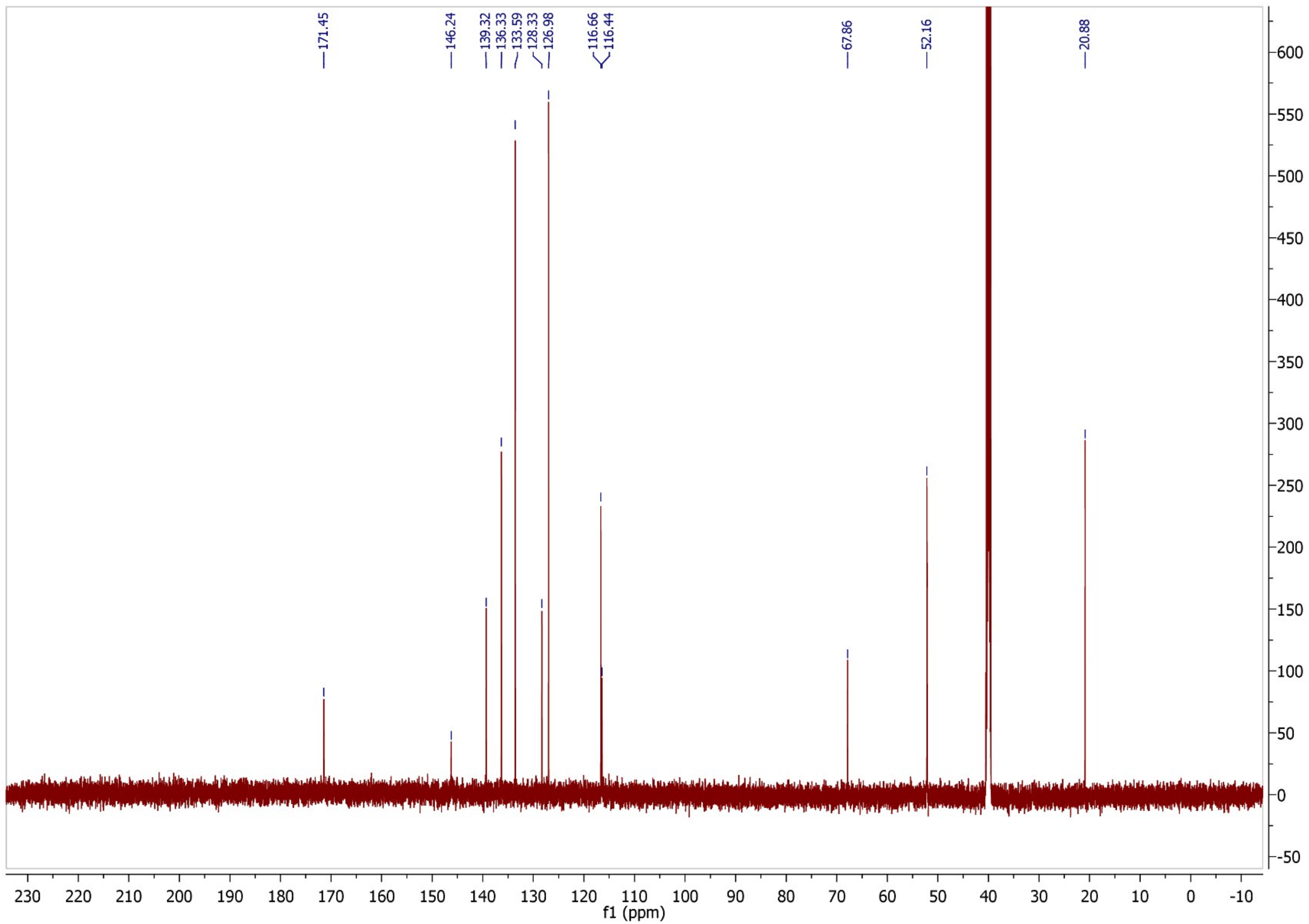

Figure S4. ^{13}C NMR spectrum of VBTMA (151MHz, DMSO).

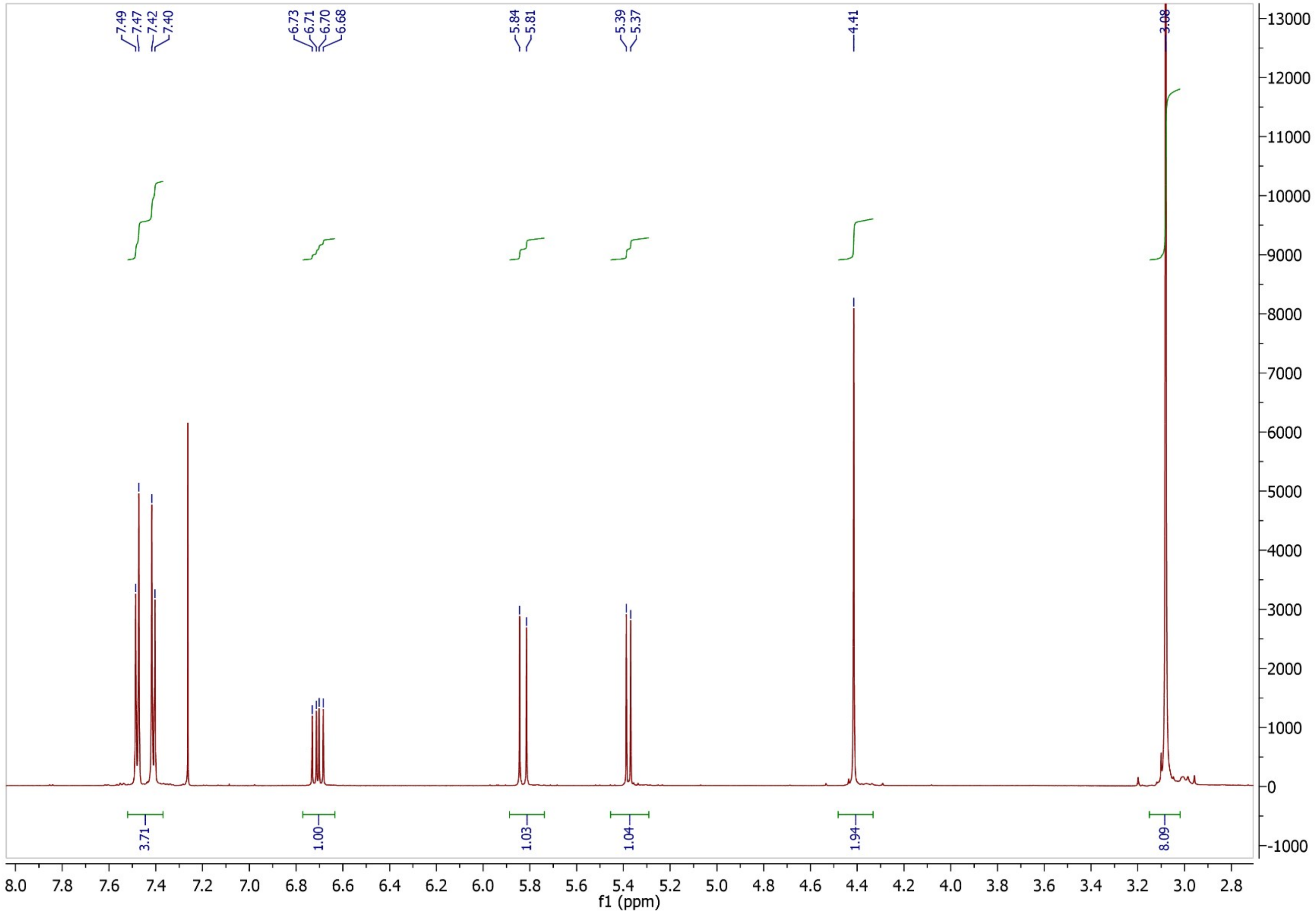

Figure S5. ^{1}H NMR spectrum of $VBTANTf_2$ (600MHz, $CDCl_3$).

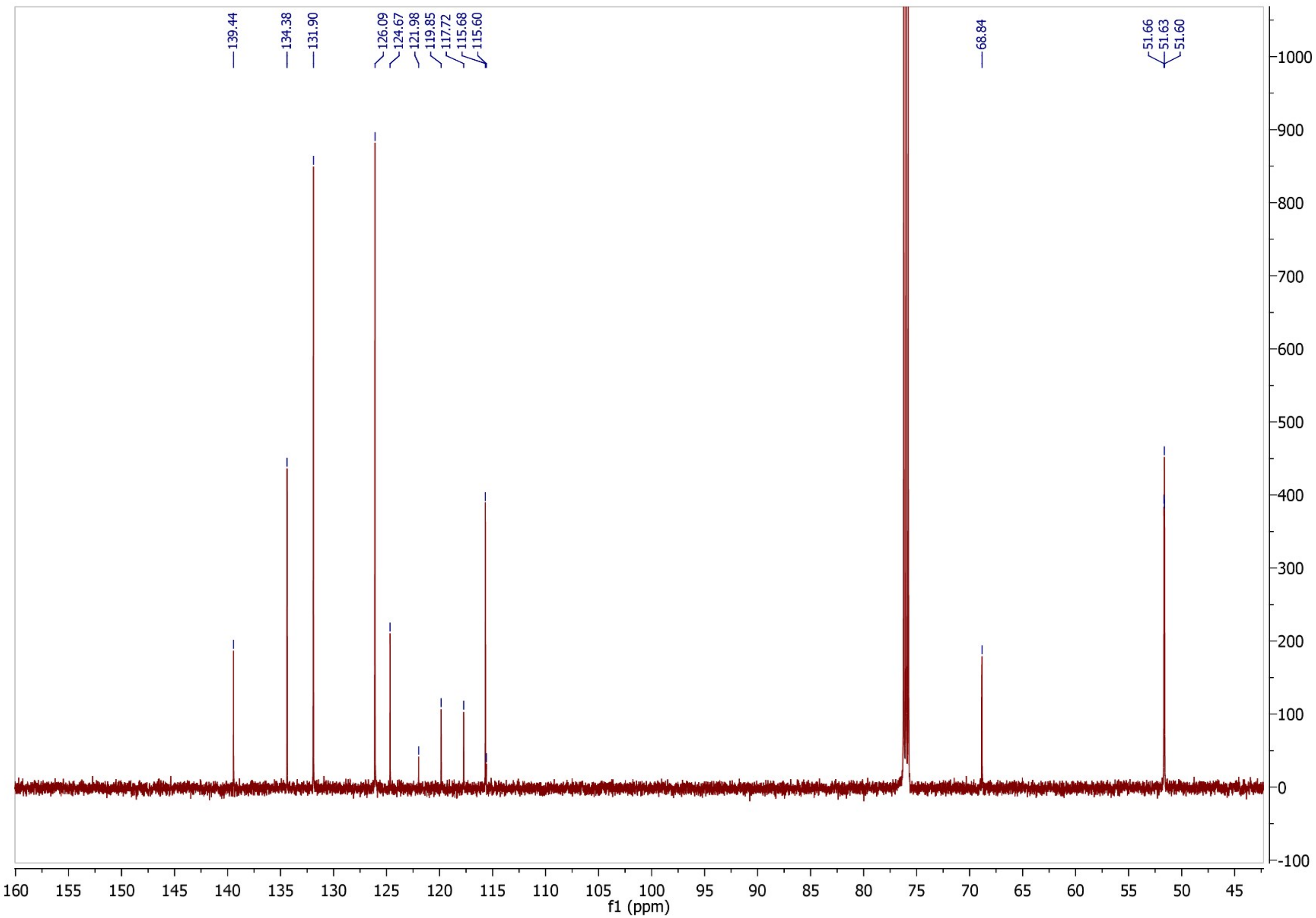

Figure S6. ^{13}C NMR spectrum of $VBTANTf_2$ (151MHz, $CDCl_3$).

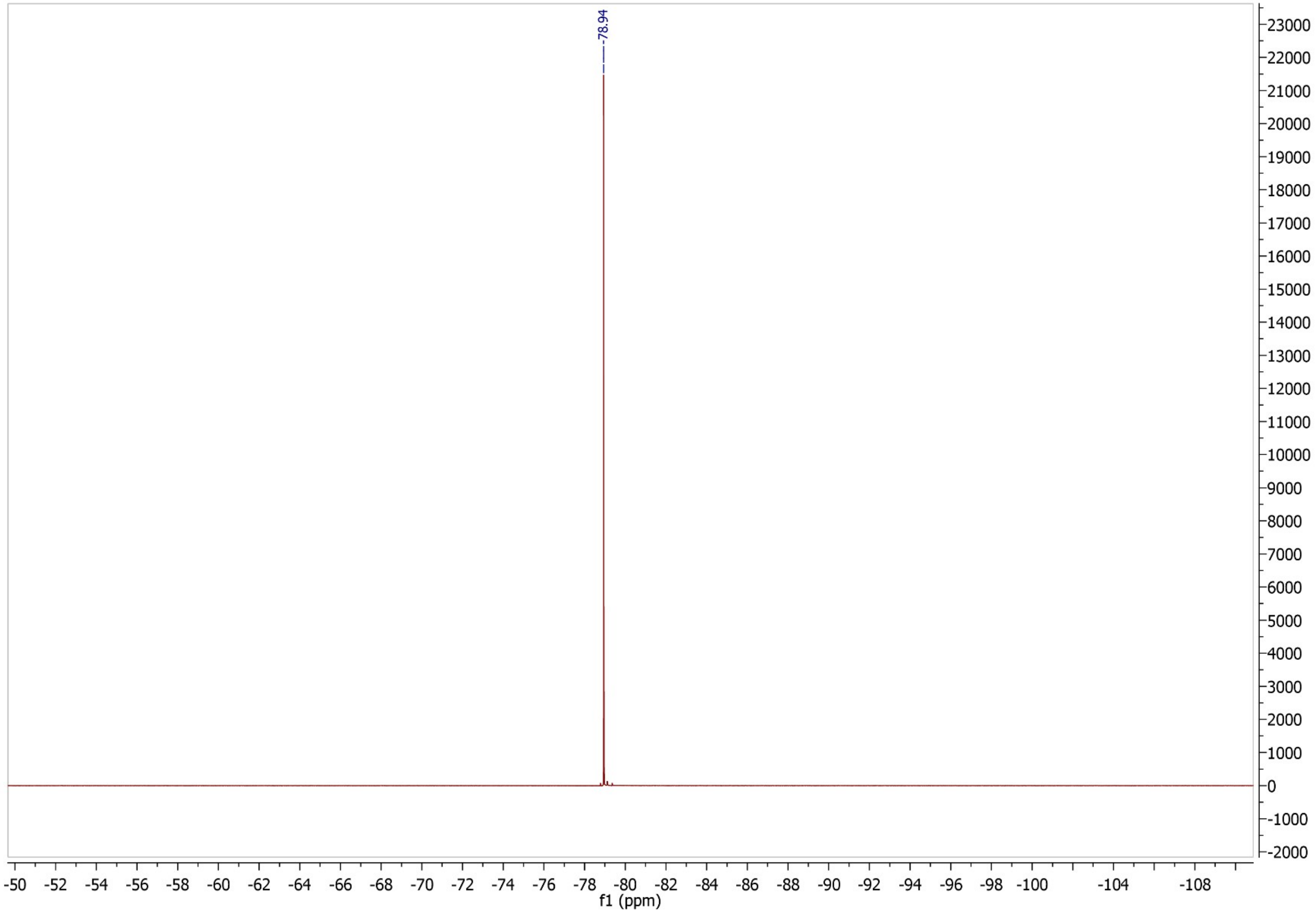

Figure S7. ^{19}F NMR spectrum of $VBTANTf_2$ (565MHz, $CDCl_3$).

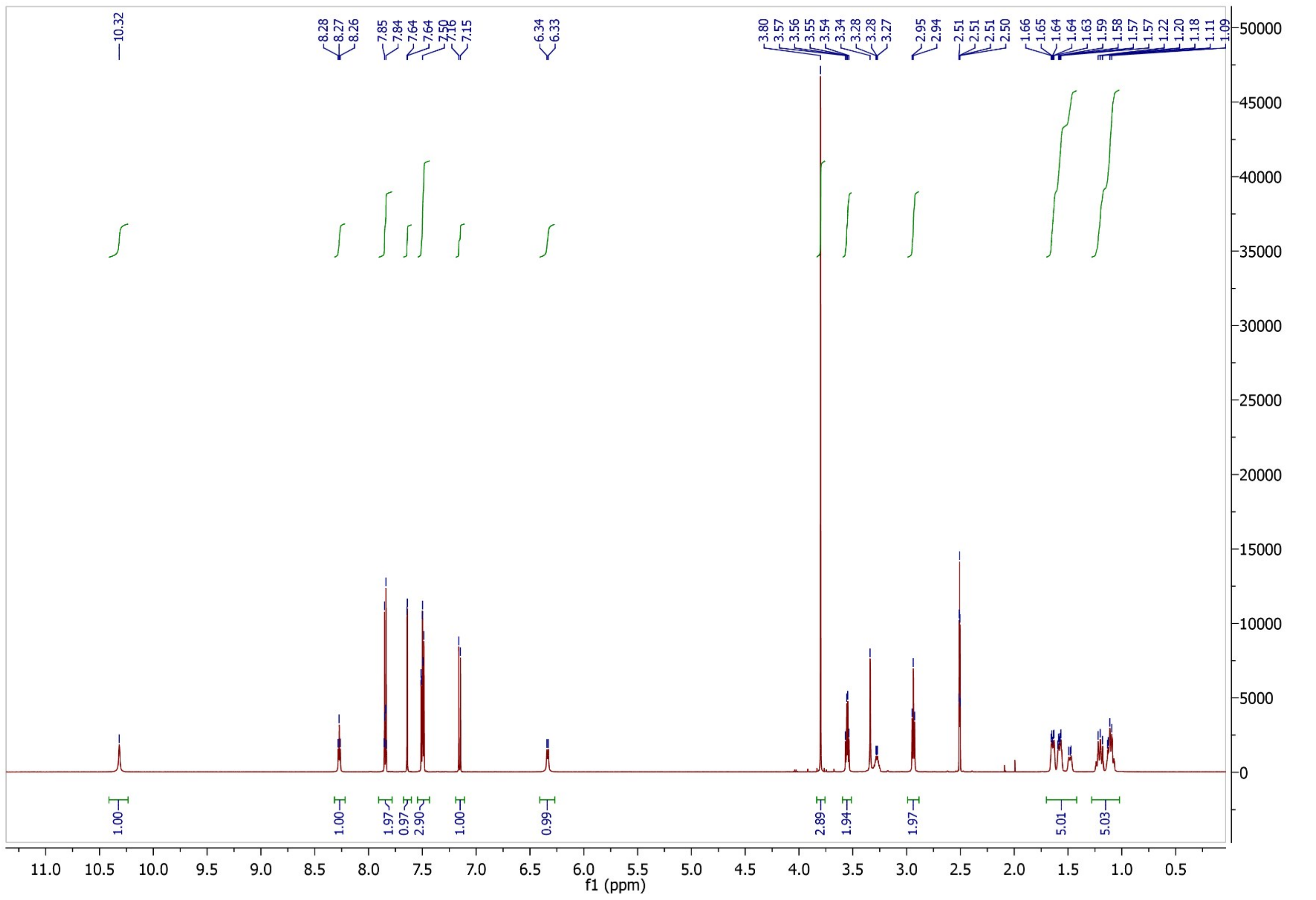

Figure S8. ^{1}H NMR spectrum of GLIB (600MHz, DMSO).

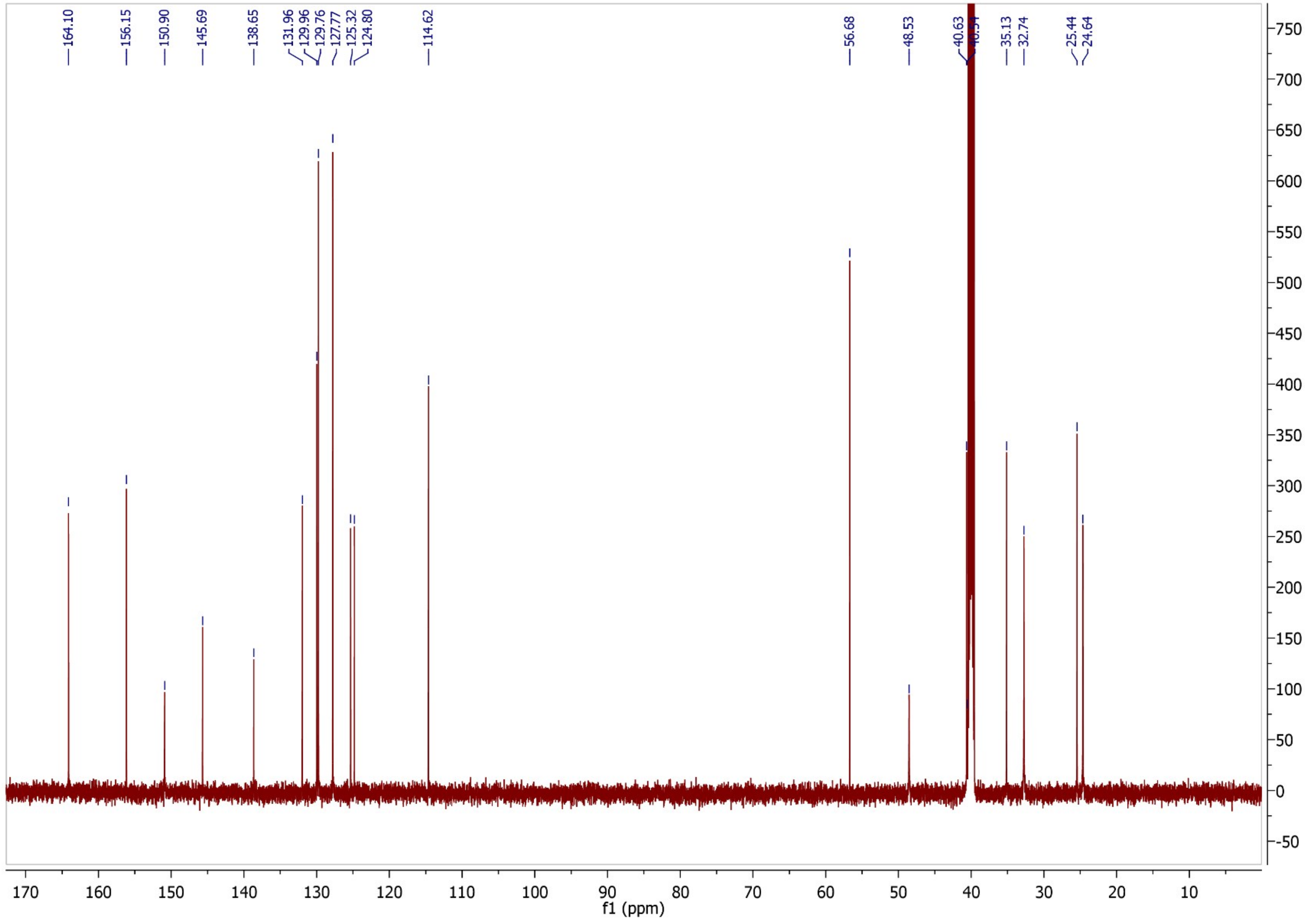

Figure S9. ^{13}C NMR spectrum of GLIB (151MHz, DMSO).

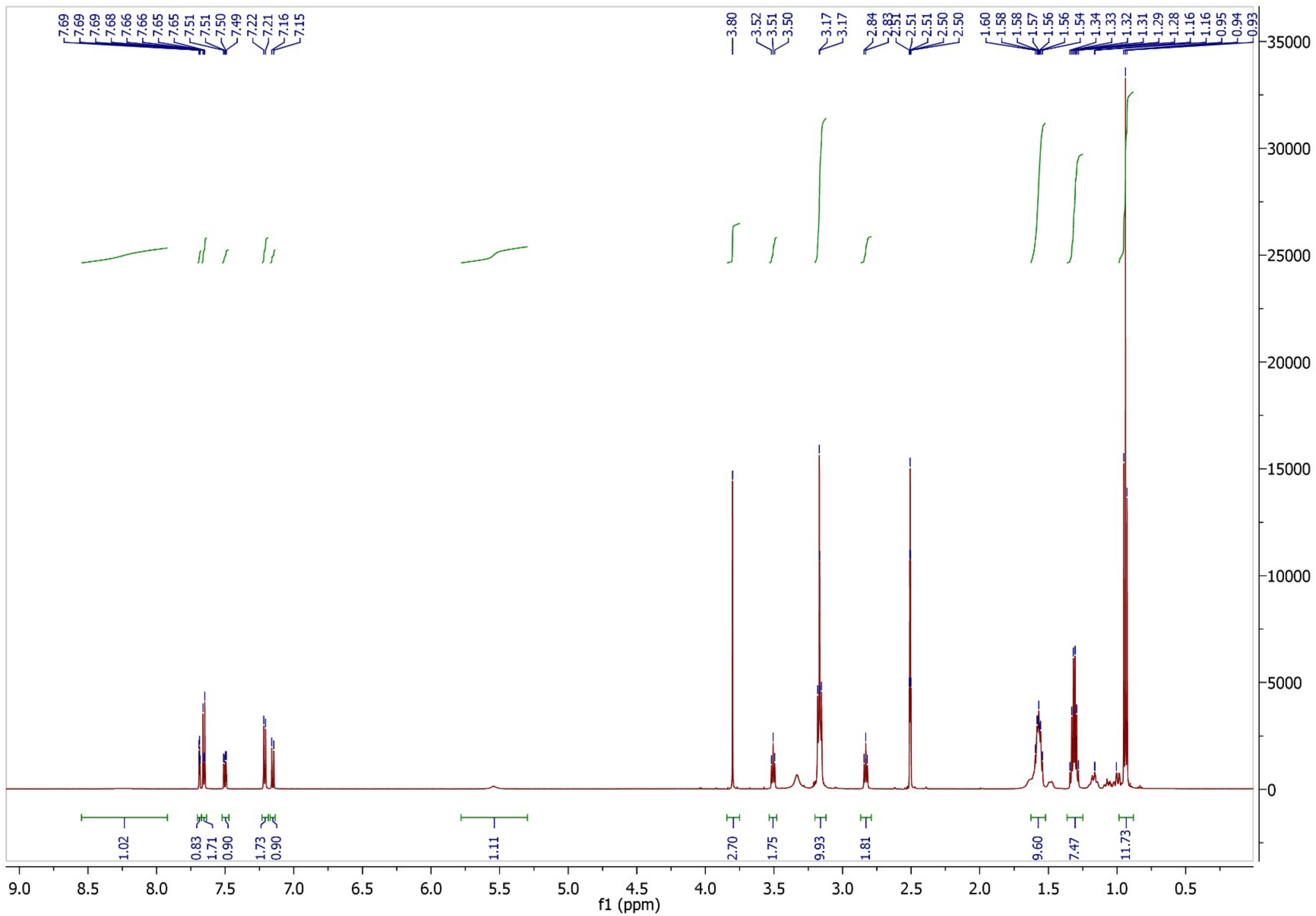

Figure S10. ^{1}H NMR spectrum of GLIB-TBA (600MHz, DMSO).

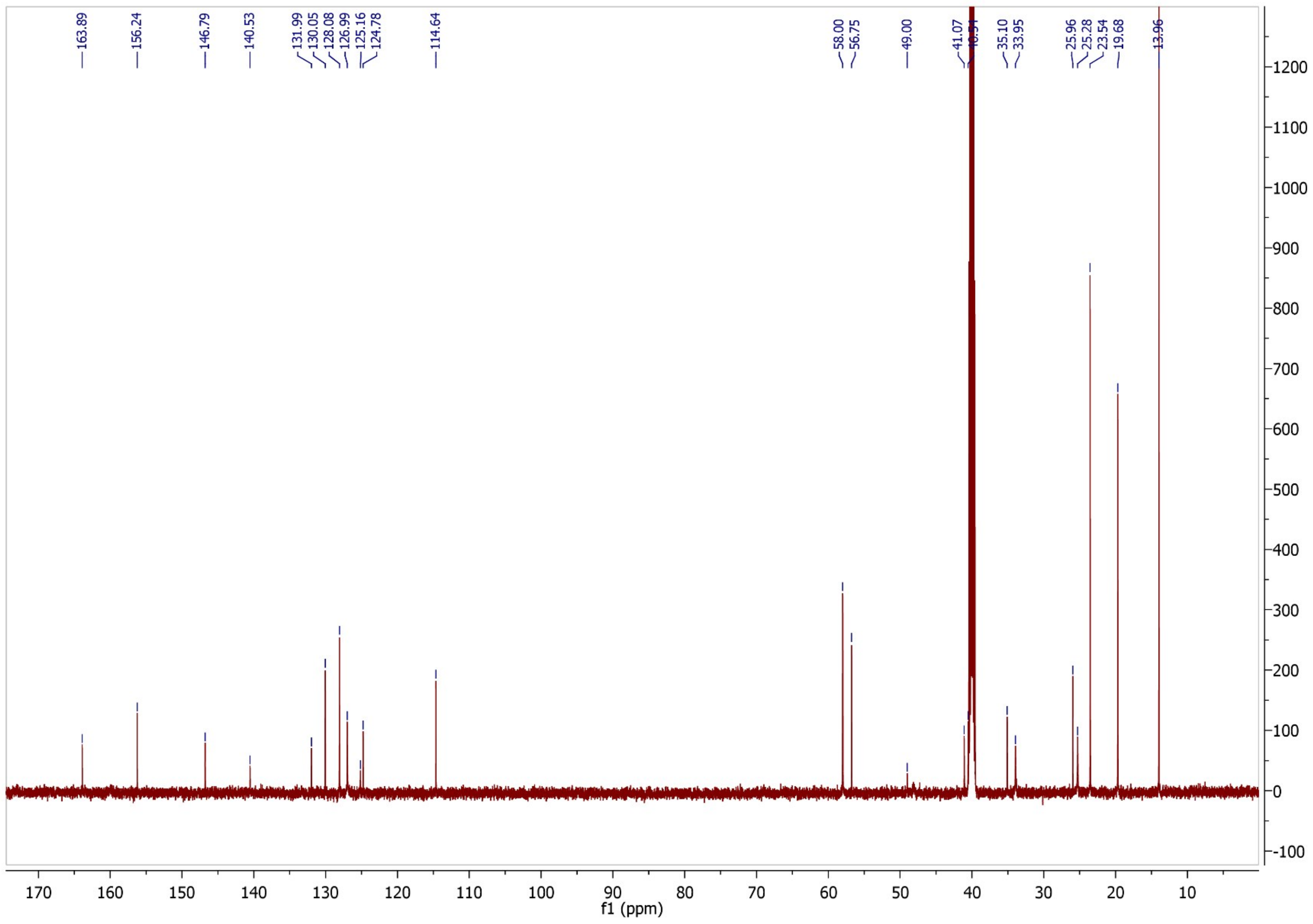

Figure S11. ^{13}C NMR spectrum of GLIB-TBA (151MHz, DMSO).

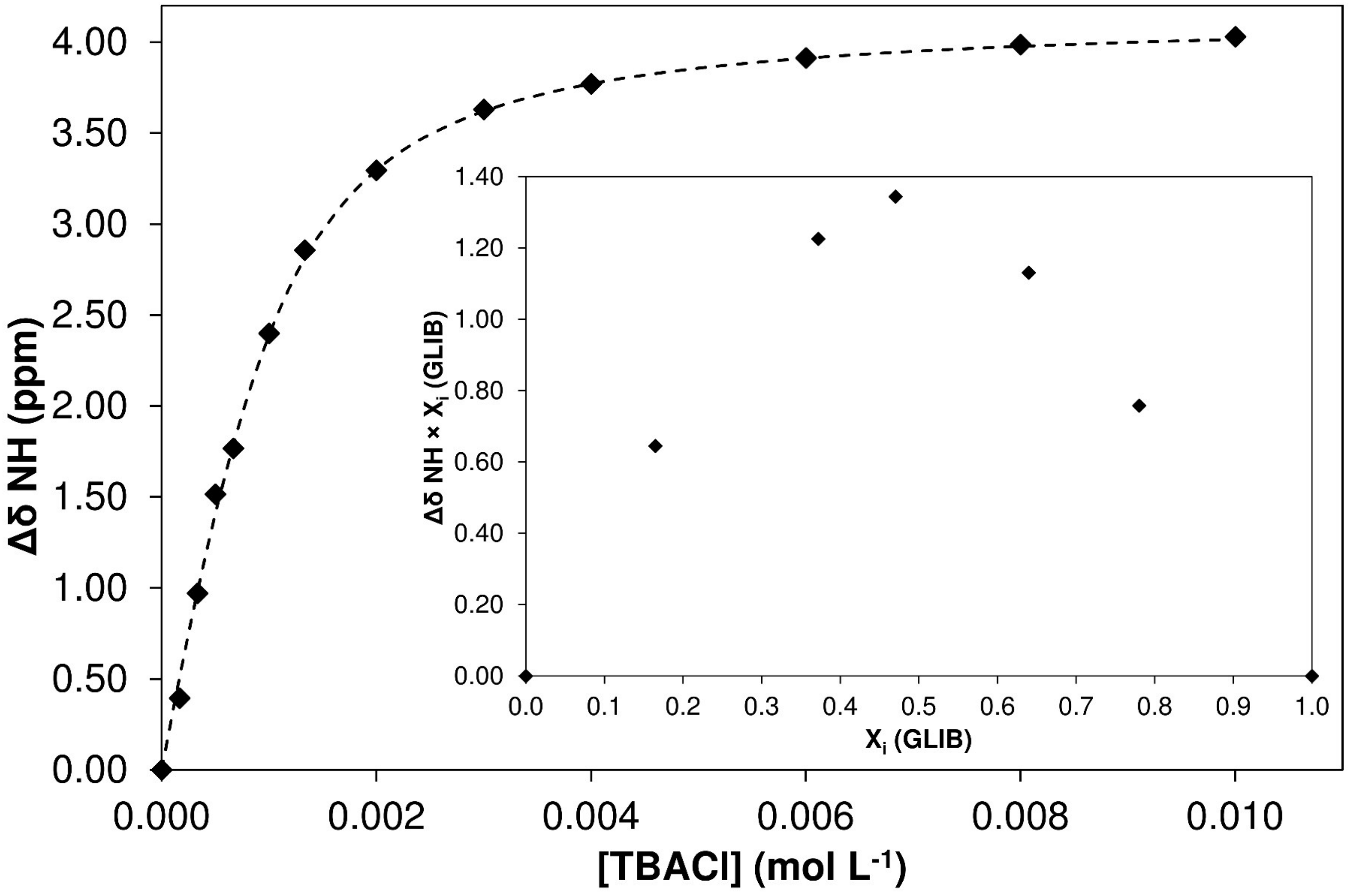

Figure S12. ^{1}H NMR titration isotherm and Job plot of GLIB *vs.* TBACl.

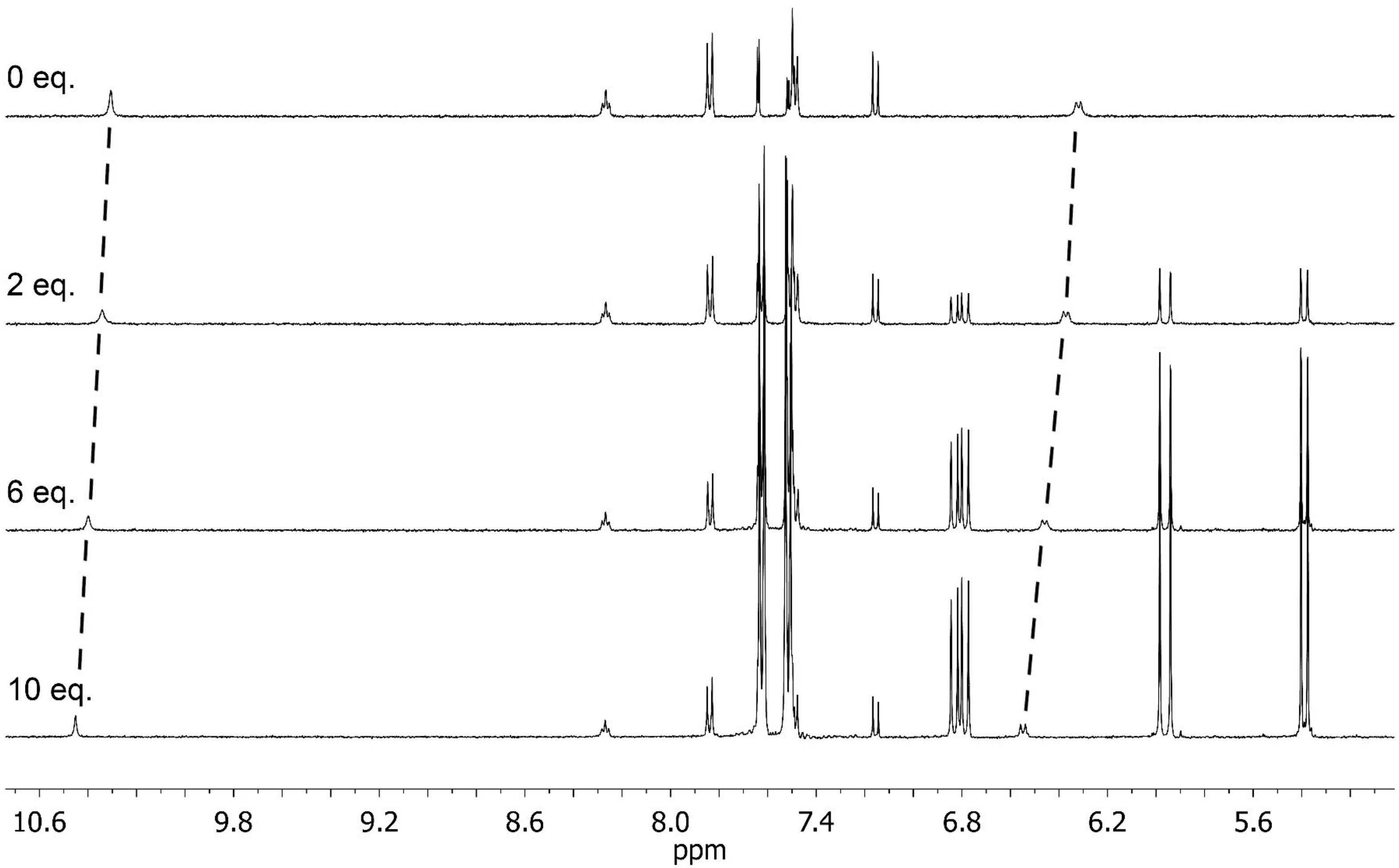

Figure S13. Overlay of ^{1}H NMR spectra collected during the titration of GLIB *vs.* VBTAC. From the top, 0 eq., 2 eq., 6 eq. and 10 eq. of VBTAC added.

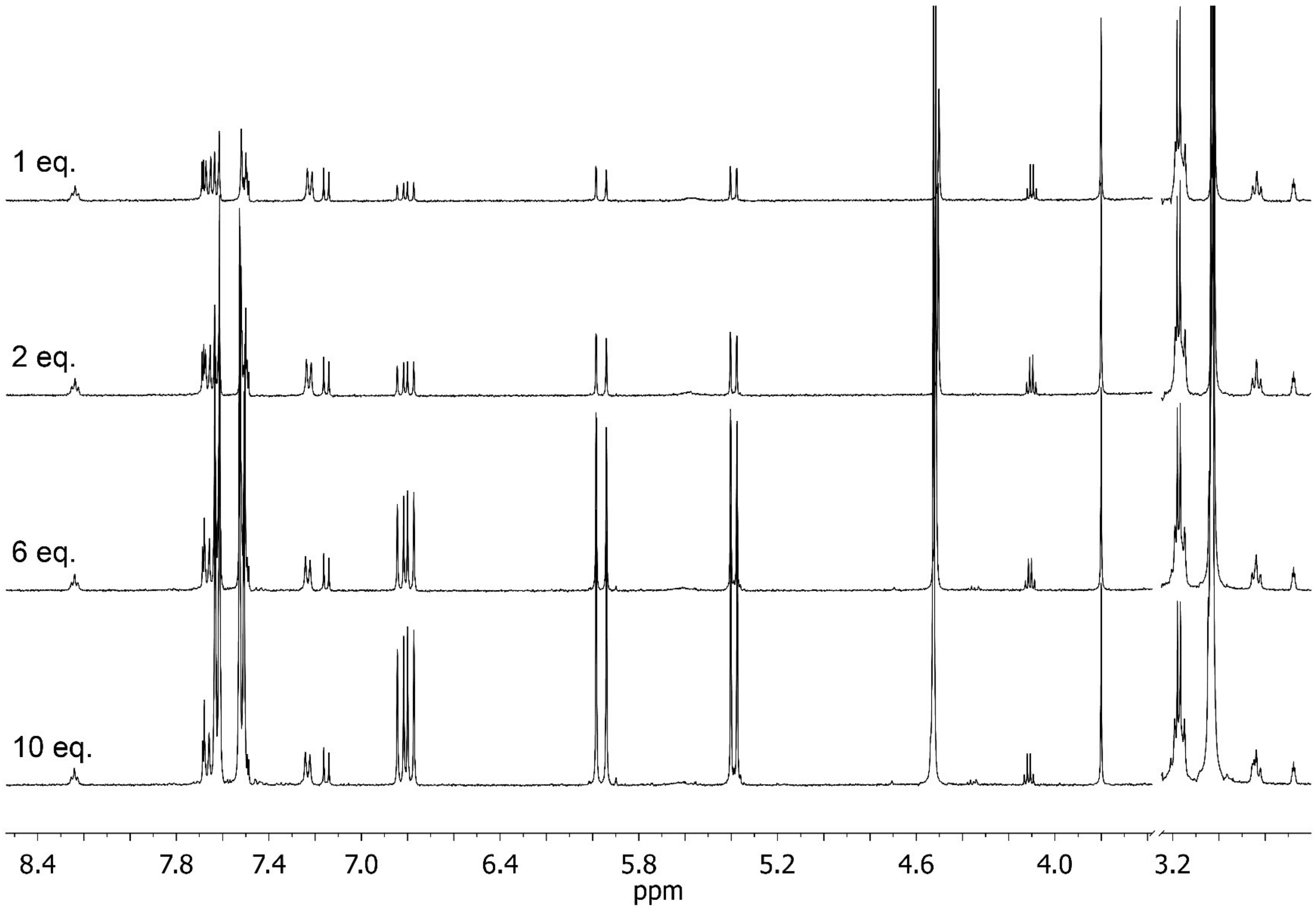

Figure S14. Overlay of ^{1}H NMR spectra collected during the titration of GLIB-TBA *vs.* VBTAC. From the top, 1 eq., 2 eq., 6 eq. and 10 eq. of VBTAC added.

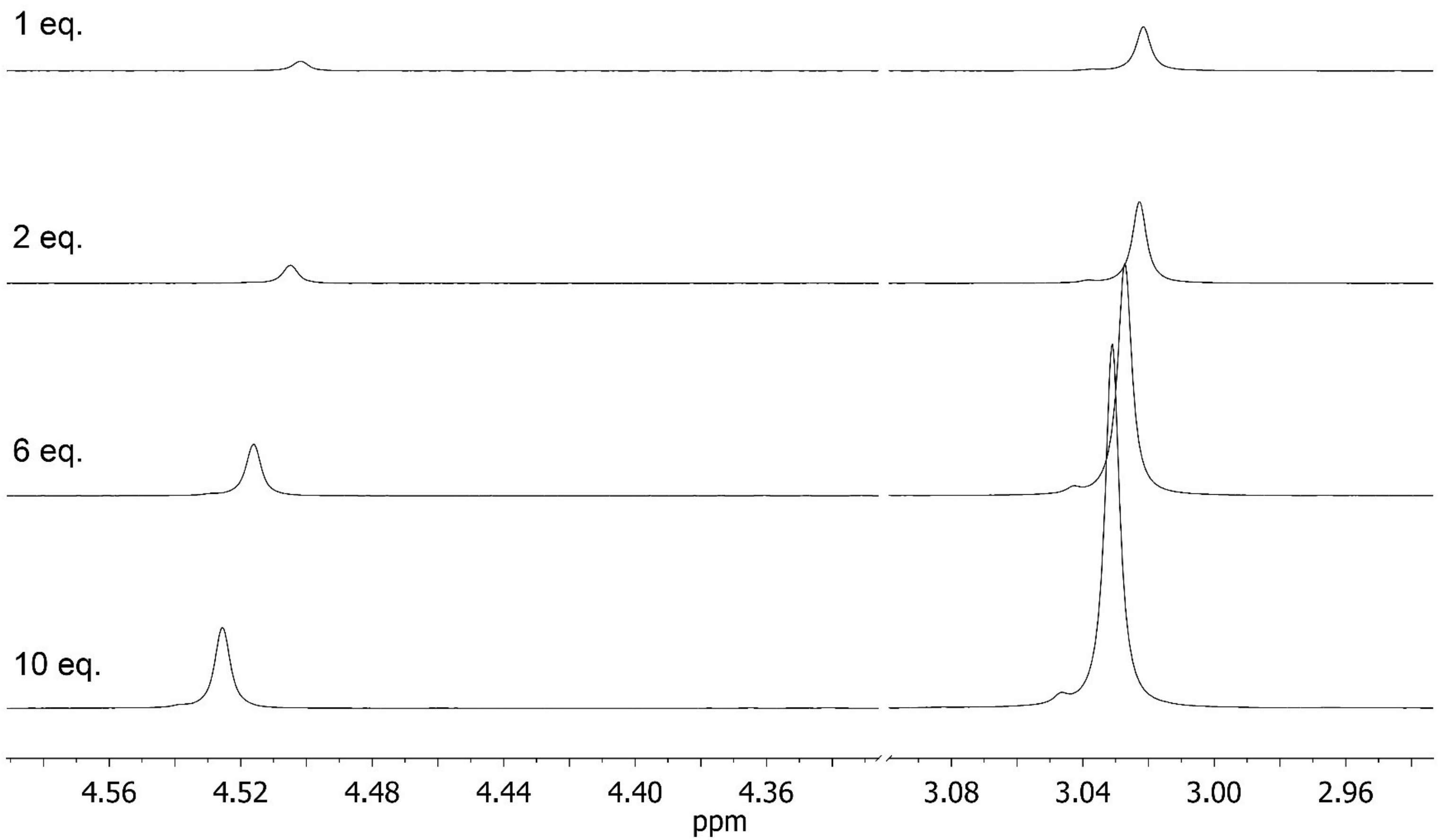

Figure S15. Characteristic signal movement during the ^{1}H NMR titration of GLIB-TBA *vs.* VBTAC. From the top, 1 eq., 2 eq., 6 eq. and 10 eq. of VBTAC added.

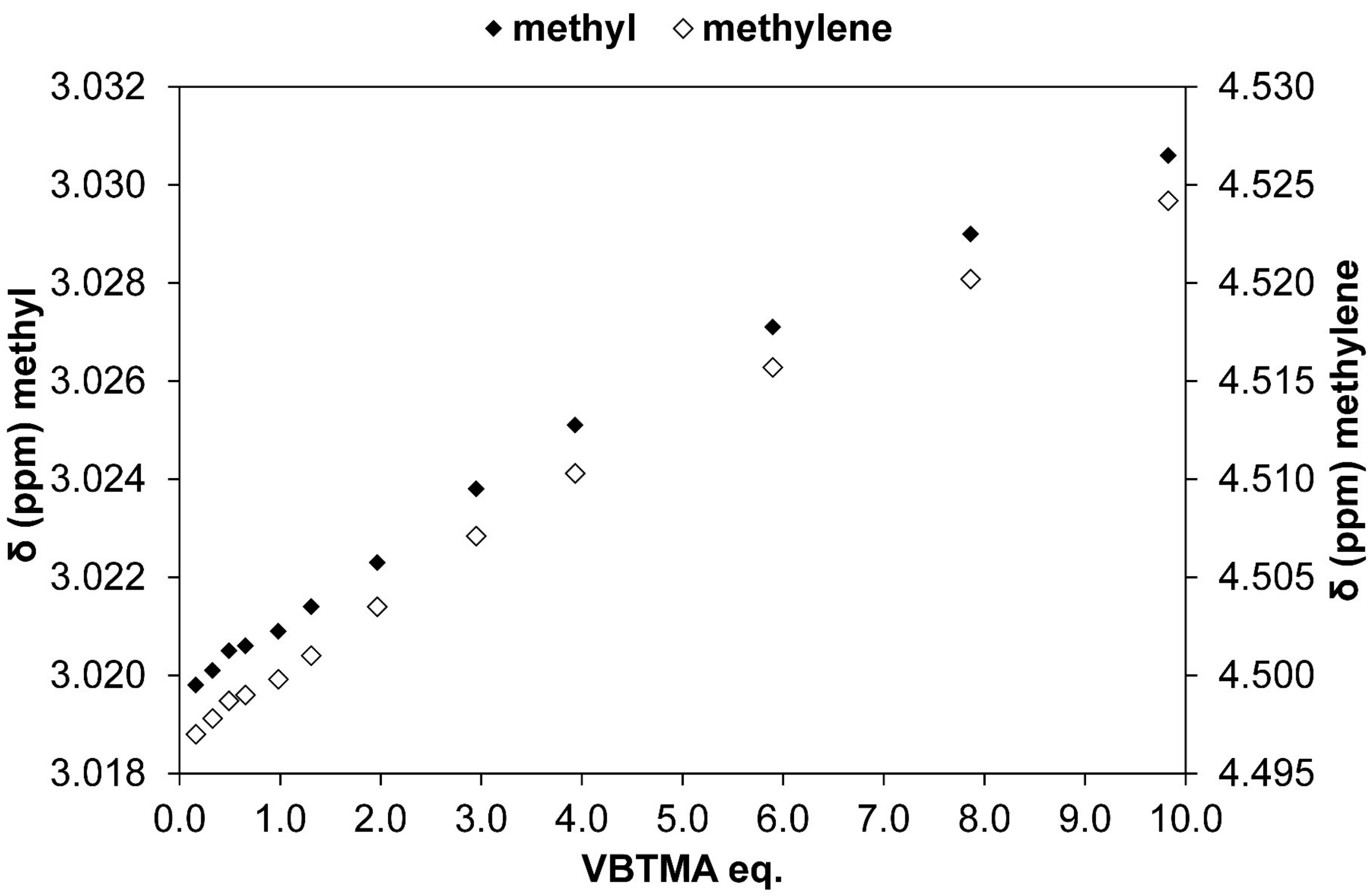

Figure S16. Change in chemical shift of the quaternary ammonium methyl groups (left axis) and methylene protons (right axis) of 4-vinylbenzyltrimethylammonium during the titration of GLIB *vs.* VBTMA.

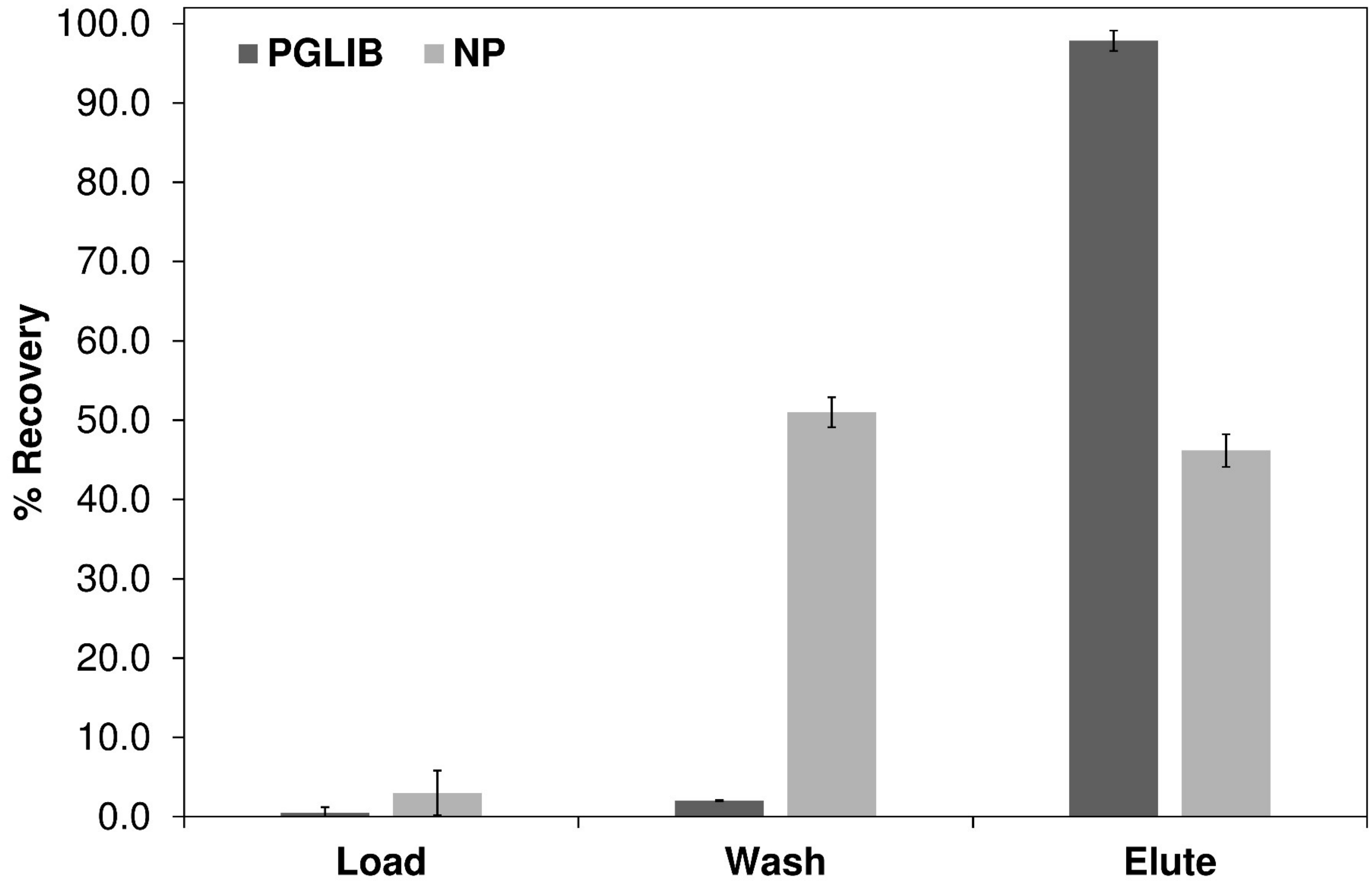

Figure S17. GLIB recovery (%) on P_{GLIB} and NP, at each step of the optimised SPE protocol.

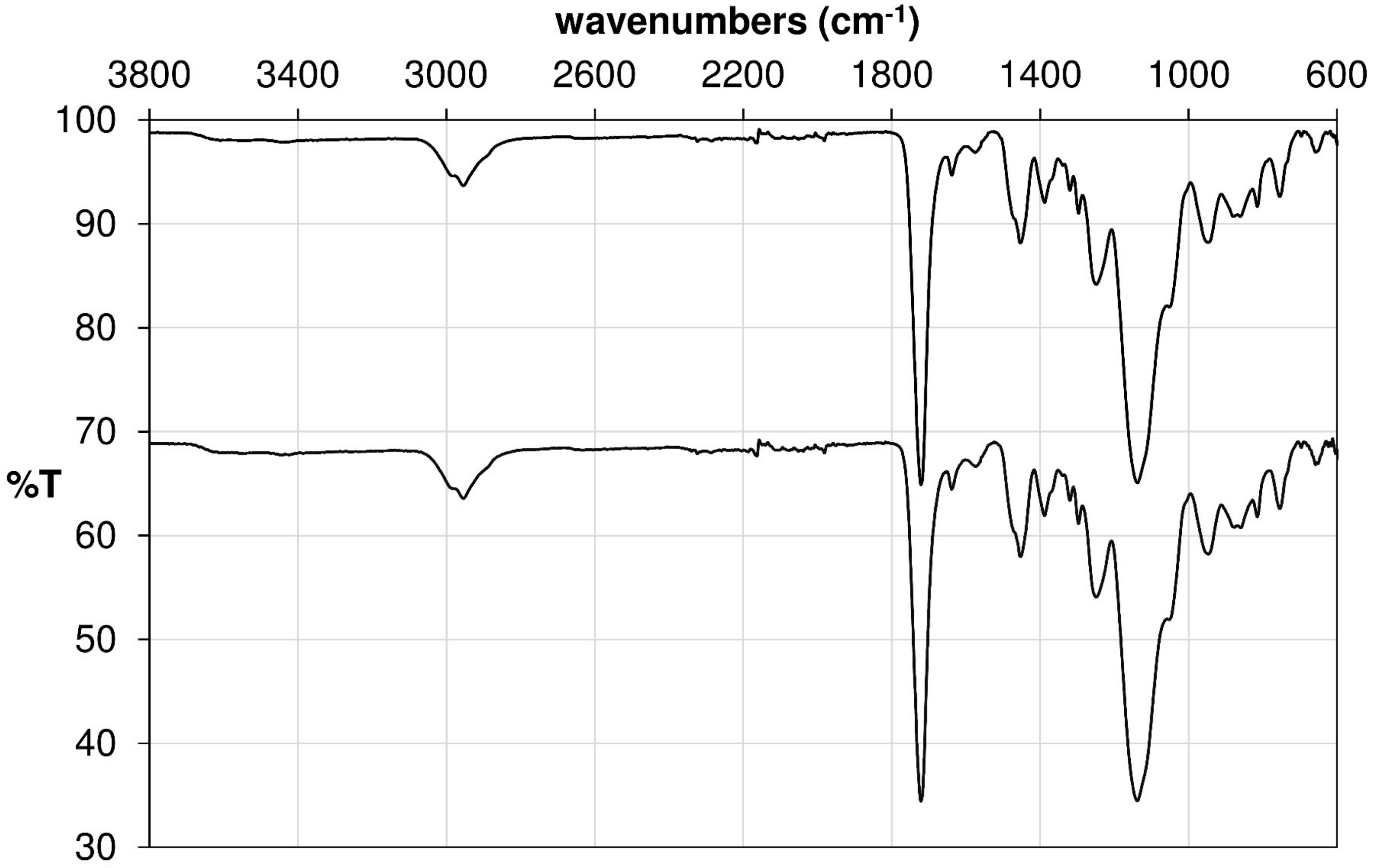

Figure S18. FT-IR spectra of P_{GLIB} (top) and NP (bottom).
